# Single-cell analysis of human basal cell carcinoma reveals novel regulators of tumor growth and the tumor microenvironment

**DOI:** 10.1126/sciadv.abm7981

**Published:** 2022-06-10

**Authors:** Christian F. Guerrero-Juarez, Gun Ho Lee, Yingzi Liu, Shuxiong Wang, Matthew Karikomi, Yutong Sha, Rachel Y. Chow, Tuyen T. L. Nguyen, Venus Sosa Iglesias, Sumaira Aasi, Michael L. Drummond, Qing Nie, Kavita Sarin, Scott X. Atwood

**Affiliations:** 1 Department of Developmental and Cell Biology, University of California, Irvine, Irvine, CA 92697, USA.; 2 Department of Mathematics, University of California, Irvine, Irvine, CA 92697, USA.; 3 NSF-Simons Center for Multiscale Cell Fate Research, University of California, Irvine, Irvine, CA 92697, USA.; 4 Center for Complex Biological Systems, University of California, Irvine, Irvine, CA 92697, USA.; 5 Department of Dermatology, Stanford University School of Medicine, Stanford, CA 94305, USA.; 6 Department of Dermatology, University of California, Irvine, Irvine, CA 92697, USA.; 7 Chao Family Comprehensive Cancer Center, University of California, Irvine, Irvine, CA 92697, USA.

## Abstract

How basal cell carcinoma (BCC) interacts with its tumor microenvironment to promote growth is unclear. We use singe-cell RNA sequencing to define the human BCC ecosystem and discriminate between normal and malignant epithelial cells. We identify spatial biomarkers of tumors and their surrounding stroma that reinforce the heterogeneity of each tissue type. Combining pseudotime, RNA velocity–PAGA, cellular entropy, and regulon analysis in stromal cells reveals a cancer-specific rewiring of fibroblasts, where STAT1, TGF-β, and inflammatory signals induce a noncanonical WNT5A program that maintains the stromal inflammatory state. Cell-cell communication modeling suggests that tumors respond to the sudden burst of fibroblast-specific inflammatory signaling pathways by producing heat shock proteins, whose expression we validated in situ. Last, dose-dependent treatment with an HSP70 inhibitor suppresses in vitro vismodegib-resistant BCC cell growth, Hedgehog signaling, and in vivo tumor growth in a BCC mouse model, validating HSP70’s essential role in tumor growth and reinforcing the critical nature of tumor microenvironment cross-talk in BCC progression.

## Teaser

Single-cell analysis of human basal cell carcinoma shows a WNT5A reactive stroma that promotes tumor HSP70 to maintain growth.

## INTRODUCTION

Basal cell carcinoma (BCC) is a locally invasive skin cancer and the most common human cancer worldwide with an estimated lifetime risk between 20 and 30% and increasing incidence rates in a number of regions including North America, Europe, Asia, and Australia (*1*). BCCs originate from inappropriate activation of the Hedgehog (HH) signaling pathway, in which secreted HH ligand binds the cholesterol transporter patched homologue 1 (PTCH1) and negates PTCH1-mediated suppression of the G protein–coupled receptor Smoothened (SMO). SMO then activates the GLI (glioma-associated oncogene homolog) family of transcription factors (TFs) to promote proliferation and tumor growth. Although the mortality rate for BCC is low, the large affected patient population imposes substantial morbidity and cost (*1*).

Although surgery remains the gold standard of therapy for BCC (*2*), it is not a practical option for tumors on cosmetically sensitive body parts or for metastatic disease. SMO inhibitors, vismodegib and sonidegib, have emerged as promising treatments for advanced disease, with a response rate of around 30% in metastatic BCC and 45% in locally advanced BCC (*2*). However, SMO mutations driving drug resistance are common, and up to 21% of patients treated with vismodegib were found to undergo tumor regrowth during treatment (*3*). Additional pathways that contribute to BCC drug resistance include phosphatidylinositol 3-kinase (PI3K)/MTOR (Mammalian target of rapamycin) (*4*, *5*), WNT (*6*), aPKC ι/λ (*7*), NOTCH1 (*8*), RAS/MAPK (*9*, *10*), and activation of MRTF (*11*, *12*), to name a few. New therapeutic options are needed to treat advanced BCC.

How stroma interacts with and promotes the growth of BCCs is unclear. Upon hierarchical clustering of cancer-associated FIB (CAF) markers in BCC, squamous cell carcinoma, and melanoma, three distinct subgroups can be stratified, each corresponding to the specific cancer type (*13*). Specifically, BCC CAFs are notable for their high expression of platelet-derived growth factor receptor β (PDGFRβ), S100A4, and TWIST. Within different histopathologic subtypes of BCCs, the tumor-to-stroma ratio is significantly divergent, with infiltrative BCCs presenting the lowest ratio (*14*). Genes coding for extracellular matrix (ECM) components are also up-regulated in BCCs, suggesting a tumor-induced remodeling of the stromal matrix (*15*). In addition, expression of stromal proteins has been shown to predict the aggressiveness of BCCs (*16*) and distinguish between infiltrative BCC and desmoplastic trichoepithelioma (*17*). Together, these studies show that expressed factors in BCC stroma can play important roles in tumor growth, angiogenesis, and metastasis. Defining BCC-stroma interactions may be a vital, yet understudied, part of tumor progression and result in more efficacious therapies.

Single-cell RNA sequencing (scRNA-seq) technologies allow the analysis of intrasample heterogeneity, tumor/sample microenvironment, pathogenic pathways, and cell-cell interactions in oncogenic contexts (*18*). Using this technology, we define BCC cellular heterogeneity, cell-cell interactions, and novel active pathways in BCC. We differentiate between malignant and normal epithelia, identify a stromal inflammatory response driven by WNT5A, characterize a subgroup of BCC keratinocytes that overexpress heat shock proteins, and provide data supporting the heat shock protein (HSP) pathway as a potential novel therapeutic target for BCC.

## RESULTS

### Resolving the cellular ecosystem of human BCC

**Fig. 1.**
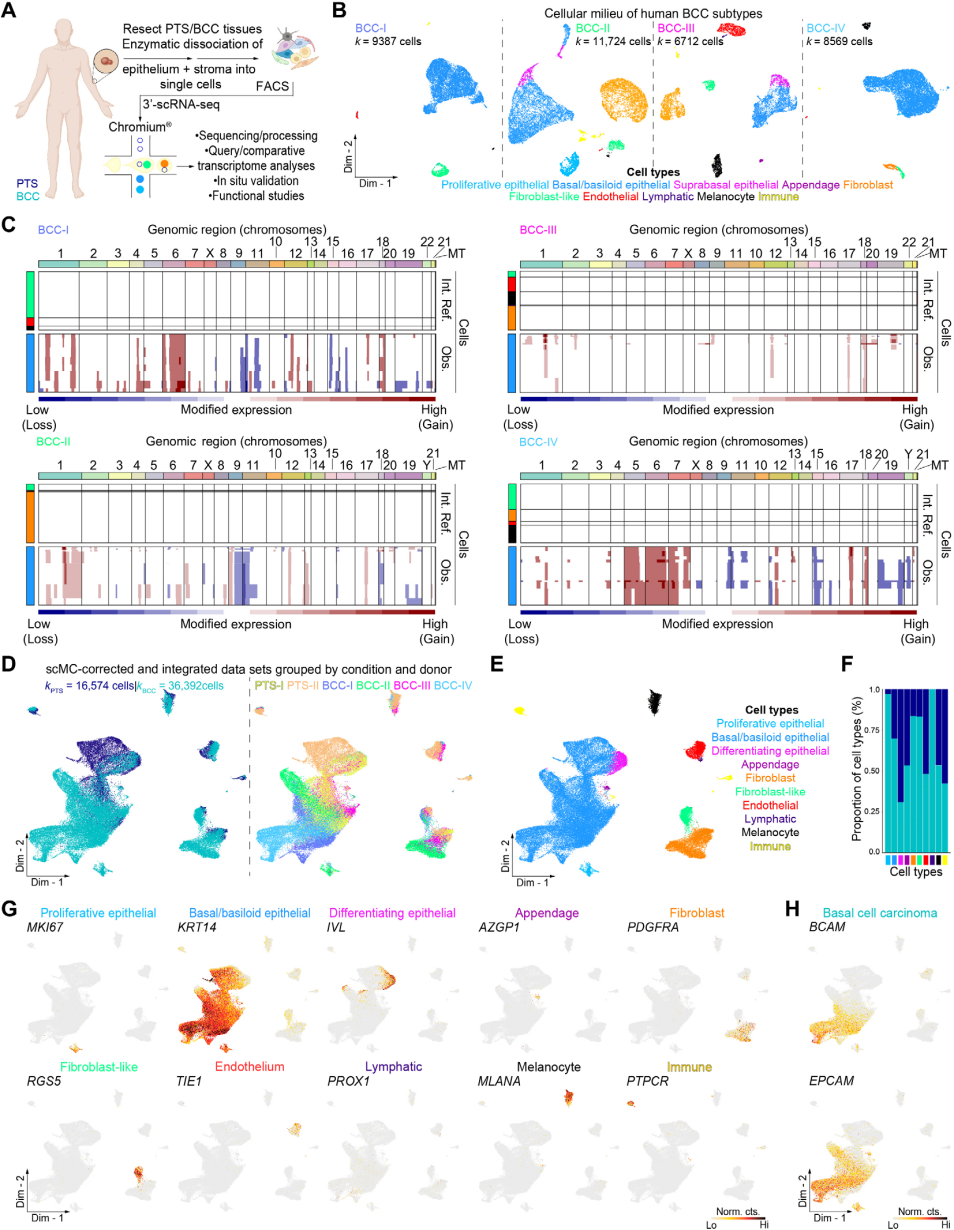
Cellular characterization of human BCC subtypes using scRNA-seq. (**A**) Schematic representation of in toto epithelial and stromal tissue isolation and processing from human PTS and BCC tissues for 3′-droplet–enabled single-cell RNA sequencing (scRNA-seq). (**B**) Two-dimensional clustering of single cells isolated from individual human BCC subtypes. IDs represent subtype and donor. BCC subtypes are color-coded on the basis of subtype and donor and include the following: superficial, nodular, and infiltrative (BCC-I); superficial and nodular (BCC-II); unknown/“hybrid” (BCC-III); and infiltrative with perineural invasion (BCC-IV). Ten distinct meta-clusters are identified at distinct proportions across BCC subtypes and annotated with their putative identities. The putative identity of each cell meta-cluster is defined on the bottom and color-coded accordingly. (**C**) Copy number variant analysis of putative malignant epithelial cells with InferCNV. Blue indicates low modified expression, corresponding to genomic loss; red indicates high modified gene expression, corresponding to genomic gain. Internal reference cells refer to nonepithelial, nonimmune control cells. Observations refer to putative malignant epithelial cells. Genomic regions (chromosomes) are labeled and color-coded. (**D**) Clustering of corrected and integrated PTS and BCC datasets is grouped by condition and donor using scMC. Conditions and donor are labeled and color-coded. (**E**) Two-dimensional clustering reveals cellular heterogeneity of integrated human PTS and BCC datasets. Ten distinct metaclusters are identified at various proportions across BCC subtypes and annotated with their putative cell type identities. The putative identity of each cell meta-cluster is defined on the right and color-coded accordingly per cell type. (**F**) Proportion of cell types grouped by condition. (**G** and **H**) Feature plots showing bona fide genes (G) and BCC-specific epithelial markers (H). Gray, low normalized gene expression based on normalized counts; black, high normalized gene expression based on normalized counts.

To resolve the cellular ecosystem of human BCCs, we sorted viable, single cells in toto from primary human BCC surgical discards (*n* = 4), including peritumor skin (PTS) tissues (*n* = 2), and subjected them to 3′-droplet–enabled scRNA-seq (Fig. 1A and fig. 1A) (*19*). The primary BCC subtypes considered in this study included superficial, nodular, and infiltrative BCC (ID: BCC-I; *k =* 9837 cells); superficial and nodular BCC (ID: BCC-II; *k* = 11,724 cells); unknown/“hybrid” BCC (ID: BCC-III; *k* = 6712 cells); and infiltrative with perineural invasion BCC (ID: BCC-IV; *k* = 8569 cells). PTS tissues constituted skin directly adjacent to BCC lesions. In total, we processed 56,162 raw single cells (*k*_PTS_ = 17,727 versus *k*_BCC_ = 38,435). After putative doublet/multiplet removal and quality control filtering of individual libraries (fig. S1B and tables S1 to S3), 52,966 “valid” cells remained (*k*_PTS_ = 16,903 versus *k*_BCC_ = 37,667). To resolve the cellular diversity present in individual tumors and enable downstream query and comparative gene expression analyses, we processed and characterized individual BCCs using Seurat (*20*) and visualized the inferred putative cell types in two-dimensional space. We identified 10 coarse-grained cell types based on bona fide biomarkers, which included *MKI67*^+^ proliferative epithelial cells, *KRT14*^+^ basal epithelial/tumor cells, terminally differentiated *IVL*^+^ keratinocytes, *AZPG*^+^ appendage-associated cells, *PDGFRA*^+^ fibroblastic cells, *RGS5*^+^ FIB-like cells, *TIE1*^+^ endothelial cells, *PROX1*^+^ lymphatic endothelial cells, *MLANA*^+^ melanocytic cells, and immune cells identified by expression of *PTPRC* (Fig. 1B). We did not confidently identify cell clusters with gene expression signatures enriched in *Stratum spinosum* keratinocytes or Schwann/neural-like cells (fig. S2).

To identify putative malignant tumor cells present in primary BCC samples, we subjected the *KRT14*^+^ epithelial/tumor cells to InferCNV analysis (InferCNV of the Trinity CTAT Project; https://github.com/broadinstitute/inferCNV). We observed aberrant genomic profiles, associated with chromosome duplication (red) and deletion (blue), in *KRT14*^+^ epithelial cells from BCC-I, BCC-II, and BCC-IV donors when compared to their counterpart nonepithelial, nonimmune internal reference cells (Fig. 1C). BCC-III did not display significant aberrant genomic structure changes when compared to other BCC subtypes. Rather, its profile resembled more those from nonappendage, *KRT14*^+^ epithelial cells present in the PTS samples (fig. S3), suggesting that some tumors do not have significant copy number variations driving tumor growth. Although InferCNV inferred aberrant genomic changes in *KRT14*^+^ epithelial cells, it cannot identify individual malignant cells.

When integrating both BCC and PTS datasets using Seurat, we noticed independent clustering of BCC *KRT14*^+^ epithelial/tumor cells from PTS, with further inter-BCC partitioning (fig. S4A). Unlike *KRT14*^+^ epithelial/tumor cells, all other nonepithelial cell types did not drift or cluster independently from each other regardless of donor. The high BCC tumor heterogeneity is in congruence with other reports indicating a high degree of transcriptome-driven epithelial, intertumoral heterogeneity in other human cancers, including melanoma and squamous cell carcinoma (*21*, *22*). To determine an alternative approach to identify BCC-associated *KRT14*^+^ epithelial/tumor cell states that significantly differ from PTS, we compared Seurat-based integration with four distinct yet widely popular clustering methodologies, including SCTransform (*23*), LIGER (*24*), Harmony (*25*), and scMC (*26*) (Fig. 1D and fig. S4). All algorithms clustered nonepithelial cells together, irrespective of condition or donor. However, Seurat, SCTransform, LIGER, and Harmony clustered epithelial cells indistinctly, irrespective of condition or donor, whereas clustering with Seurat was driven entirely by donor, making it difficult to identify and interpret BCC-specific epithelial cell types or states (fig. S4). In sharp contrast, scMC clustered BCC and PTS epithelial cells distinctly while maintaining clustering of transcriptionally similar cell types (Fig. 1D). As scMC retains biological variation while removing technical variation associated with each sample, we therefore used the resultant scMC-corrected BCC-PTS data for downstream query and comparative analysis.

scMC maintained the same 10 distinct cell types found by independent BCC clustering with Seurat (Fig. 1, E to G). Quantification of each coarse-grained cell type partitioned by condition and donor revealed relative cell type frequency similarities across BCC and PTS (Fig. 1F). Non-PTS *KRT14*^+^ epithelial/tumor cells uniquely expressed known BCC-associated gene biomarkers including *BCAM* and *EPCAM* (Fig. 1H) (*27*). *KRT14*^+^ epithelial/tumor cells from BCC-III showed a “hybrid” position where many cells significantly overlapped with the PTS samples, whereas other cells uniquely clustered singly, matching our previous observations with InferCNV analyses (Fig. 1, C and D). In sum, our benchmarking approach and comparative scRNA-seq clustering analyses resolved the distinct cellular landscape of human BCCs and revealed major *KRT14*^+^ epithelial cell type differences compared to PTS, suggesting a high level of inter- and intratumor transcriptional heterogeneity between human BCC samples.

### Defining normal versus malignant epithelial cells

**Fig. 2.**
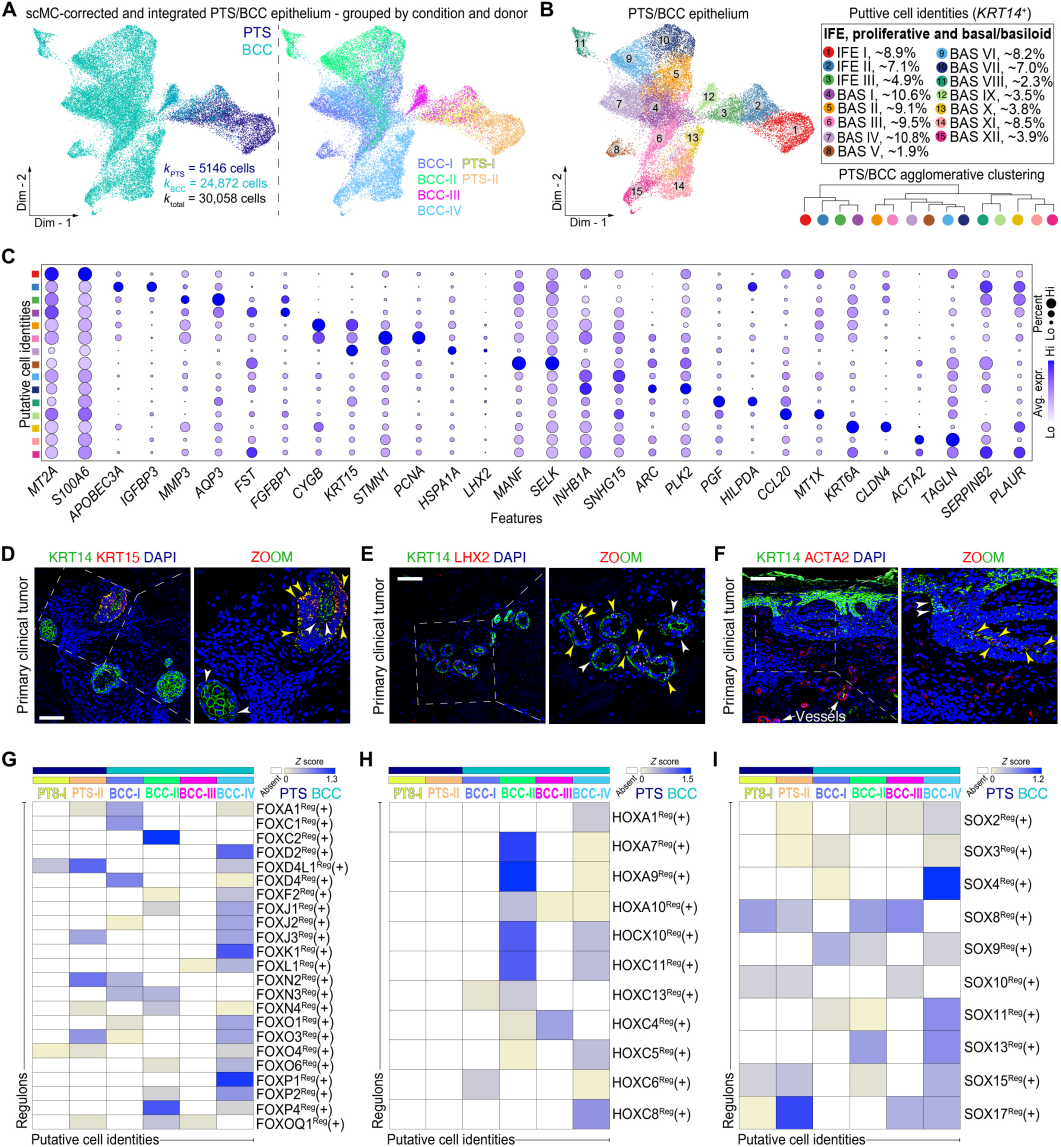
Comparison of epithelial cells reveals regulators of malignancy in human BCC. (**A** and **B**) Clustering of 30,058 corrected *KRT14*^+^ epithelial cells from human PTS and BCC subtypes grouped by condition and donor. Fifteen putative *KRT14*^+^ epithelial cell identities, including 1 proliferating epithelial and 3 interfollicular epithelial cells, and 11 basal/basaloid epithelial cells were identified and defined. PTS/BCC agglomerative clustering shows relationships between *KRT14*^+^ epithelial cells. Cells are color-coded accordingly. (**C**) Dot plot of top two marker genes identified by differential gene expression among epithelial cells. Gray, low average gene expression; purple, high average gene expression. Size of circle represents the percentage of cells expressing gene markers of interest. (**D** to **F**) Protein immunostaining of select BCC–epithelial cell markers shows cluster specificity and distinct spatial localization in human primary clinical tumors. Inset shows magnified area of BCC nest. White arrows point at epithelial cells expressing KRT14. Yellow arrows point at epithelial cells coexpressing protein of interest and KRT14. Tissues were counterstained with DAPI. Scale bars, 100 μm. (**G** to **I**) Heatmap of condition and donor-specific active gene regulatory networks demonstrates differentially active FOX, HOX, and SOX regulons in BCC epithelial cells compared to PTS (*Z* score > 0). Yellow, low regulon activity; blue, high regulon activity; white, absent regulon activity.

To define the epithelial/tumor cellular landscape of human BCC and PTS samples, we subclustered 30,058 *KRT14*^+^ epithelial-derived cells (*k*_PTS_ = 5146 versus *k*_BCC_ = 24,872) and identified 15 coarse-grained epithelial cell clusters, all defined by expression of unique gene biomarkers (Fig. 2, A to C). Three of the subpopulations (IFE I to III) appear to be normal epithelia and make up nearly all the PTS samples and a small proportion of the BCC samples, whereas the rest of the cells cluster uniquely to the BCC-associated samples (BAS I to XII). This is distinct from what was found in squamous cell carcinoma, where most tumor keratinocytes were indistinguishable from normal keratinocytes except for the presence of a small population of tumor-specific keratinocytes (*22*). Whether the overlap in normal and tumor keratinocytes in squamous cell carcinoma is due to the clustering algorithm used, inter-/intratumoral heterogeneity, or a large proportion of normal keratinocytes in the tumor samples is unclear. To explore their gene expression profile and spatial architecture, we spatially resolved select gene products, including *KRT15* (BAS II), *LHX2* (BAS IV), and *ACTA2* (BAS XI). KRT15 marked a subset of KRT14^high^ tumor nests (Fig. 2D), LHX2 marked the nucleus of cells along the outer periphery of KRT14^high^ tumor nests (Fig. 2E), and ACTA2 marked the outer periphery of KRT14^low^ tumor nests (Fig. 2F). *LHX2* is significantly expressed in bulk-level RNA-seq data from vismodegib-sensitive and vismodegib-resistant advanced BCC tumors compared to normal skin with *ACTA2* showing tumor-specific variability and *KRT15* not showing significance (*28*), reinforcing the heterogeneity of BCC tumors and highlighting how single-cell data can resolve significantly expressed genes that are otherwise averaged out in bulk-level RNA-seq studies.

We next defined the identity of “transformed” cells by scoring individual *KRT14*^+^ cells with a previously defined BCC-associated gene expression profile that includes coexpression of *EPCAM*, *BCAM*, and *TP63* (*27*, *29*). When grouped by condition and qualitatively and quantitatively evaluated, we observed that most of the BCC-associated cells expressed some of these markers, but not all three. Of interest, *EPCAM* and *BCAM* were rather unique to BCC, whereas *TP63* was lowly expressed in PTS samples, a similar pattern to bulk-level RNA-seq data where *EPCAM* and *BCAM* showed significant expression in BCC tumors and *TP63* was significant in only a subset of tumors (*28*). Canonical HH target genes such as *PTCH1*, *GLI1*, and *GLI2* were not reliable markers of BCC-associated cells, likely due to their lower expression levels and the limitations of the 10X Genomics platform—which relies on the chemistry used and mainly capturing highly expressing mRNAs. To develop a better measure of transformation, we identified prominent gene expression differences between BCC and PTS epithelial cells (fig. S5A). This approach led to the identification of *LGALS1* as a gene that is highly up-regulated in BCC epithelial cells (fig. S5B). LGALS1 has been previously implicated in pancreatic ductal adenocarcinoma (*30*), clear cell renal cell carcinoma (*31*), cervical cancer (*32*), and malignant melanomas (*33*). However, to our knowledge, it has not been previously implicated in BCC biology or previously identified as a marker of BCCs and is not significantly enriched in bulk-level RNA-seq data of BCC (*28*). We conducted a similar approach to identify genes associated with the different BCC samples in our cohort of donors. We identified *MYLK*, *CALM5*, *SCGB2A2*, and *KRT19* as highly expressed within each tumor sample (fig. S5C), all of whom are not significantly enriched in bulk-level RNA-seq of BCC (*28*).

To identify BCC-specific gene regulators (regulons) that may be driving condition-specific gene expression changes in the different epithelial-derived cell populations, we performed gene regulatory network (GRN) analysis using pySCENIC (*34*) and identified significantly active regulons specific to BCC subtypes in our cohort of donors (Fig. 2, G to I). In addition to identifying active regulons known to be implicated in the initiation and progression of BCC, including as GLI1^Reg^(+) and GLI2^Reg^(+) (fig. S5, D to F), we identified several classes of regulons of particular interest that include the FOX, HOX, and SOX family of TFs (Fig. 2, G to I). FOX TFs, which are highly active in BCC-IV, have been implicated in HH signaling in other systems. For instance, FOXC1, which is active in BCC-I, can activate SMO-independent HH signaling in basal-like breast cancer, suggesting that it may regulate BCC drug resistance (*35*). The HOX TFs, which are highly active in BCC-II, are main players in murine digit patterning, where HOX TFs can activate *Shh* transcription, with Shh protein establishing additional *Hox* expression zones (*36*). However, the interplay between HOX TFs and HH signaling in cancer is unclear. Last, the SOX TFs, which are highly active in BCC-IV, have several known family members with connections to BCC, including SOX2 (*37*) and SOX9 (*38*). These results suggest that there are specific regulons that are active in BCC-associated epithelial cells whose activity differs between BCC subtypes, reinforcing the heterogeneity of BCC and which may be important in BCC biology.

We were interested in using scRNA-seq data to determine whether we could resolve genes or gene-specific loci identified in other bulk-level genomic or transcriptomic studies with individual cells within the BCC macroenvironment. As a proof of principle, we used our human BCC scRNA-seq data and overlaid expression of genes associated with BCC risk loci identified via GWAS (genome-wide association study) in BCC (fig. S6) (*39*). We successfully identified several differentially expressed genes that were associated with specific BCC risk loci and that were expressed only in BCC-IV epithelial cells, including *BNC2*, *CUX1*, *ZBTB10*, and *CASC15*, whereas other genes showed broader expression across cell types in both normal and tumor cells, such as *LPP*, *PLIN2*, *HLA-B*, and *NEU1*. We also used a similar approach to identify significantly enriched vismodegib-resistant genes from bulk-level RNA-seq analysis of advanced BCC (*28*) that are expressed only in BCC epithelial cells (fig. S7, A and B). We found a cohort of genes that were nonspecific and had broad expression in other cell types in both normal and tumor contexts, including *SLC39A14* and *DUSP10*. In contrast, other genes displayed unique expression in BCC-IV epithelial cells, including *FBN3* and *SH3GL3* (fig. S7C). This approach could enable the identification of genes specific to certain BCC subtypes or BCC epithelial subclusters.

### RNA velocity analyses show distinct cellular dynamics in BCC

As BCCs display both inter- and intratumor heterogeneity, we performed RNA velocity analysis using scVelo (*40*) to better estimate and generalize transient cell states within *KRT14*^+^ epithelial cells through dynamical modeling. Coupling RNA velocity vectors with Markovnikov root and terminal states demonstrates that superficial and nodular (BCC-I); superficial, nodular, and infiltrative (BCC-II); and the unknown/“hybrid” subtype BCC (BCC-III) velocity vectors point toward a terminal state associated with high levels of BCC-associated signature genes and high in HH and WNT pathway genes (fig. S8, A to C). In contrast, the infiltrative with perineural invasion BCC (BCC-IV) displayed vectors pointing away from a region high in HH genes (fig. S8D). Velocities derived from cells with a clear high late differentiation gene signature (*41*) in BCC-I and BCC-II suggest a potential dedifferentiation fate choice of late differentiation epithelial cells in favor of a more basal-like fate in BCC (fig. S8, A and B), in contrast to normal epithelia that display velocities going toward the high late differentiation gene signature (fig. S8C). These results may reflect distinct tumor states that are also seen as a consequence of drug treatment (*6*, *42*).

### FIB heterogeneity and function in human BCC

**Fig. 3.**
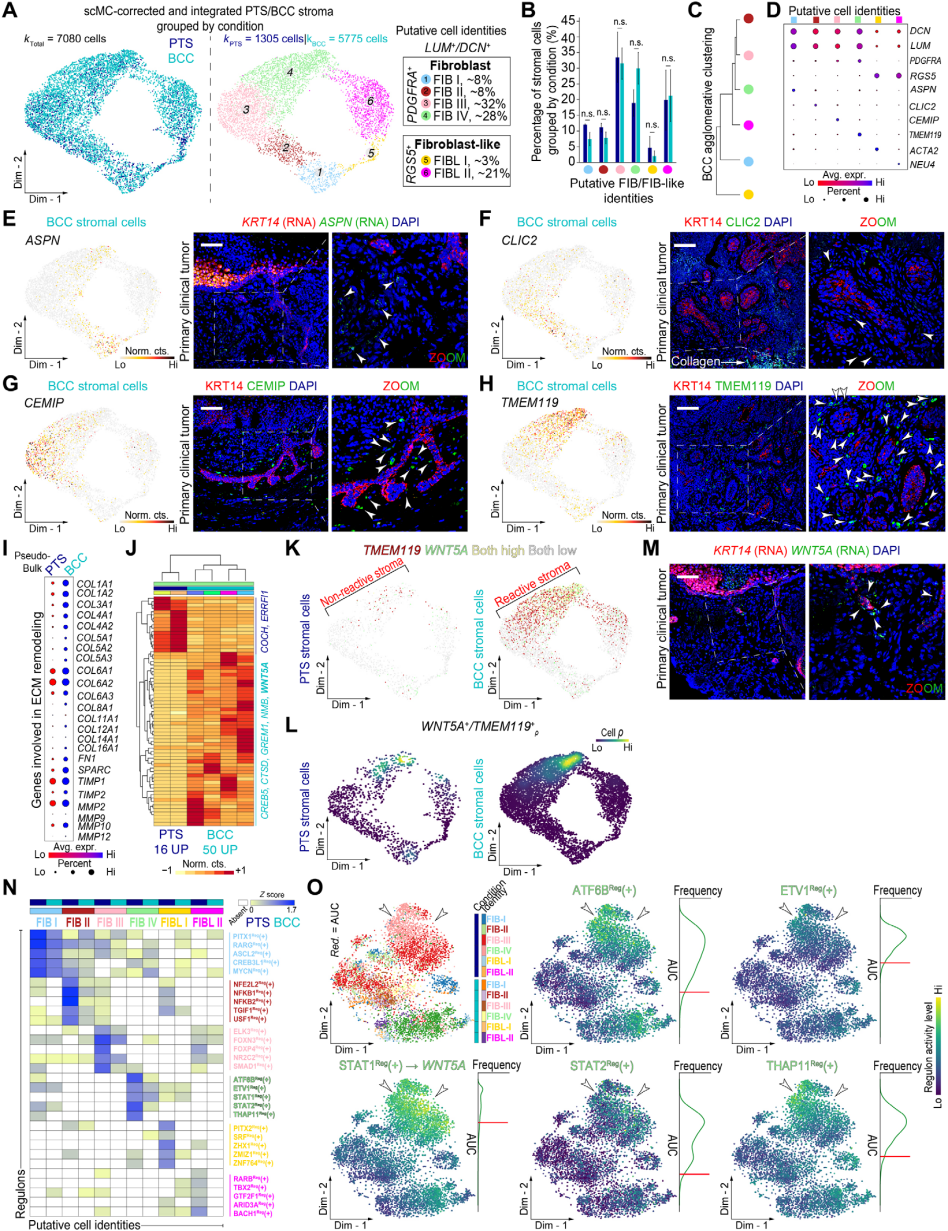
Analysis of stromal cells highlights FIB and FIB-like cell heterogeneity in human BCC. (**A**) Clustering of 7080 corrected FIB/FIB-like (FIB/FIB-like) cells from human PTS and BCC subtypes grouped by condition and subtype. Four putative *PDGFRA*^+^ FIB and two putative *RGS5*^+^ FIB-like cell identities were identified and defined. (**B** and **C**) Quantification and agglomerative clustering of FIB/FIB-like cells. Bar graph represents cell average per donor per cluster ± SEM. Unpaired Student’s two-tailed *t* test. n.s., not significant. (**D**) Dot plots of canonical/marker genes in FIB/FIB-like cells. Blue, low-average gene expression; red, high-average gene expression. (**E** to **H**) Feature plots and in situ RNA/protein staining show FIB marker specificity/distinct spatial localization in human primary clinical tumors. Inset shows magnified area in BCC nests. White arrows point at FIBs expressing gene/protein of interest. Tissues were counterstained with KRT14 (RNA/protein) and DAPI. Scale bars, 100 μm. (**I**) Pseudo-bulk dot plots of ECM remodeling genes. Red, low-average gene expression; blue, high-average gene expression. Size of circle, percentage of expressing cells. (**J**) Heatmap of differentially expressed genes in FIB IV cells. Yellow, down-regulated genes; red, up-regulated genes. (**K** and **L**) Gene expression (K) and cellular density (L) plots of *TMEM119*, *WNT5A*, or *TMEM119*;*WNT5A* cells. Purple, low cellular density; yellow, high cellular density. (**M**) RNA in situ hybridization of *WNT5A* in human primary clinical tumors. Inset shows magnified area of BCC cells. White arrows point at *WNT5A*^+^ FIBs. Tissues were counterstained with *KRT14* and DAPI. Scale bars, 100 μm. (**N** and **O**) Heatmap showing active regulons in FIB/FIB-like cells (*Z* score > 0). Yellow, low regulon activity; blue, high regulon activity; white, absent regulon activity. Regulon activity was used for dimensionality reduction in a two-dimensional embedding. White arrows mark BCC-specific FIBs (IV). Purple, low regulon activity; yellow, high regulon activity; density plots, AUC distribution per regulon.

Recent studies have identified a large degree of functional heterogeneity in fibroblasts (FIBs) and fibroblast-like (FIB-like) cells across different states and conditions in human (*21*, *22*, *43*) and mouse (*44*, *45*) skin tissues with important biological relevance in homeostasis, injury-mediated repair and regeneration, disease, and cancer. To discern whether cellular and spatial FIB or FIB-like heterogeneity exists in human BCC and PTS regions, we subclustered FIB and FIB-like cells based on expression of *PDGFRA* and *RGS5*, yielding a total of 7080 cells (*k*_PTS_ = 1305 versus *k*_BCC_ = 5775) (Fig. 3, A and B). Both cell types were collectively positive for ECM proteins *DCN* and *LUM*. This subclustering approach led to the identification of four coarse-grained FIB populations, and two FIB-like populations, all defined by differential expression of unique gene biomarkers (Fig. 3, B to D).

To explore their gene expression profile and spatial architecture in human BCC, we spatially resolved their distribution in situ using RNA in situ hybridization or protein immunostaining coupled with high-resolution confocal imaging. Cluster 1 fibroblasts (FIB I) represent ~8% of all FIBs analyzed and collectively express *ASPN* (Fig. 3E). ASPN overexpression has been shown to lead to cancer progression and enhanced metastasis, and its expression is similar in mesenchymal stromal cells and CAFs (*46*). In situ, ASPN^+^ FIBs appeared ubiquitously yet sparsely throughout the dermis (Fig. 3E). Cluster 2 FIBs (FIB II) represent ~8% of all FIBs and collectively express *CLIC2* (Fig. 3F). In situ, CLIC2^+^ FIBs are located sparsely surrounding KRT14^+^ tumor cell nests (Fig. 3F). Cluster 3 FIBs (FIB III) represented ~32% of all FIBs and collectively express *CEMIP* (Fig. 3G). In colorectal cancer, hypoxia-mediated overexpression of CEMIP in submucosa epithelial cells leads to eventual enhanced cell migration status (*47*). In addition, CEMIP^+^ FIBs surround KRT14^+^ tumor cell nests (Fig. 3G). Last, cluster 4 FIBs (FIB IV) represent ~28% of all FIBs and robustly express *TMEM119* (Fig. 3H). TMEM119 is up-regulated in osteosarcoma cells, and its overexpression is associated with increased tumor size, clinical stage, distant metastasis, and poor prognosis (*48*). Most of the TMEM119^+^ FIBs appeared to be positioned peripherally and juxtaposed to KRT14^+^ tumor nests (Fig. 3H), to a greater extent than those observed for CLIC2^+^ and CEMIP^+^ FIBs or sparse ASPN^+^ FIBs (Fig. 3, E to G). We also identified two types of *RGS5*^+^ FIB-like cells, expressing *ACTA2* (~3%) and *NEU4* (~21%). Quantification of cells from each putative FIB and FIB-like subtype partitioned by condition revealed similar cell type frequencies across BCC and PTS samples, with the exception of *TMEM119*^+^ FIBs, which appeared slightly expanded in BCC compared to PTS (Fig. 3B). Our in situ imaging analysis suggests that TMEM119^+^ FIBs segregate distinctly across KRT14^+^ tumor nests in terms of both position and density, further reinforcing the notion that significant intertumoral FIB heterogeneity exists in human BCC and that this particular population may be functionally and structurally positioned to support tumoral growth and progression.

We then examined genes coding for ECM-related proteins and compared their expression profiles between conditions to approximate the level of ECM remodeling in BCC compared to PTS. In general, we identified prominent changes in extent and expression of genes coding for various collagens, including *COL1A1, COL1A2*, *COL3A1*, *COL4A1*, *COL4A2*, *COL5A1*, *COL5A2*, *COL5A3*, *COL6A1*, *COL6A2*, *COL6A3*, *COL8A1*, *COL12A1*, *COL14A1*, and *COL16A1* (Fig. 3I). Analogous to collagen-coding genes, other ECM-related protein-coding genes, including *FN1*, *SPARC*, *TIMP1*, *TIMP2*, *MMP2*, *MMP9*, *MMP10*, and *MMP12*, were also enriched in BCC compared to PTS stroma (Fig. 3I). The expression of these ECM-related coding genes was not restricted to individual FIB subsets, but rather represents a pan-BCC ECM-related remodeling gene profile. This comparative analysis suggests a large degree of ECM-related remodeling in BCCs compared to PTS that is likely driven by expression of collagen- and metalloproteinase-coding genes.

### Rewiring FIBs to a reactive stroma state

Because TMEM119^+^ FIBs segregated distinctly across KRT14^+^ tumor nests and were significantly higher in proportion in BCC samples, we wondered whether they may have a unique gene expression profile that may functionally support tumoral growth and progression. To shed light on this notion, we performed differential gene expression analysis on cluster 4 FIBs across BCC and PTS conditions using a modified version of DEseq2 specifically tailored for single-cell analysis (*49*). This analysis led to the identification of 16 genes differentially up-regulated in PTS cluster 4 FIBs and 50 genes differentially up-regulated in BCC cluster 4 TMEM119^+^ FIBs (Fig. 3J). One particular gene, *WNT5A*, was overexpressed in BCCs and coexpressed with *TMEM119*, and its RNA localization showed a similar pattern of distribution to TMEM119 protein expression (Fig. 3, H and K to M). WNT5A has emerged as an important molecule involved in cancer progression, and recent studies have demonstrated that WNT5A regulates cancer cell invasion, metastasis, metabolism, and inflammation (*50*). Hence, our results suggest a potential functional signaling network of *TMEM119*^+^ FIBs with *KRT14*^+^ tumor cells driven through paracrine noncanonical WNT signaling.

To identify BCC-specific regulons that may be driving condition-specific gene expression changes in the different FIB populations, including *TMEM119*^+^ FIBs, we performed GRN analysis with pySCENIC and identified significantly active regulons that were specific to each FIB/FIB-like cluster in BCCs but not in PTS (Fig. 3N). The top five regulons active in *TMEM119*^+^ FIBs included ATF6B^Reg^(+), ETV1^Reg^(+), STAT1^Reg^(+), STAT2^Reg^(+), and THAP11^Reg^(+) (Fig. 3O). Our single-cell GRN analysis suggests that there are specific regulons that are active in FIB and FIB-like cells, and they differ significantly in activity and regulation of specific targets between BCC and PTS. Furthermore, we found that the STAT1^Reg^(+) regulon may be involved in the upstream regulation of the noncanonical WNT ligand *WNT5A* (Fig. 3O).

**Fig. 4.**
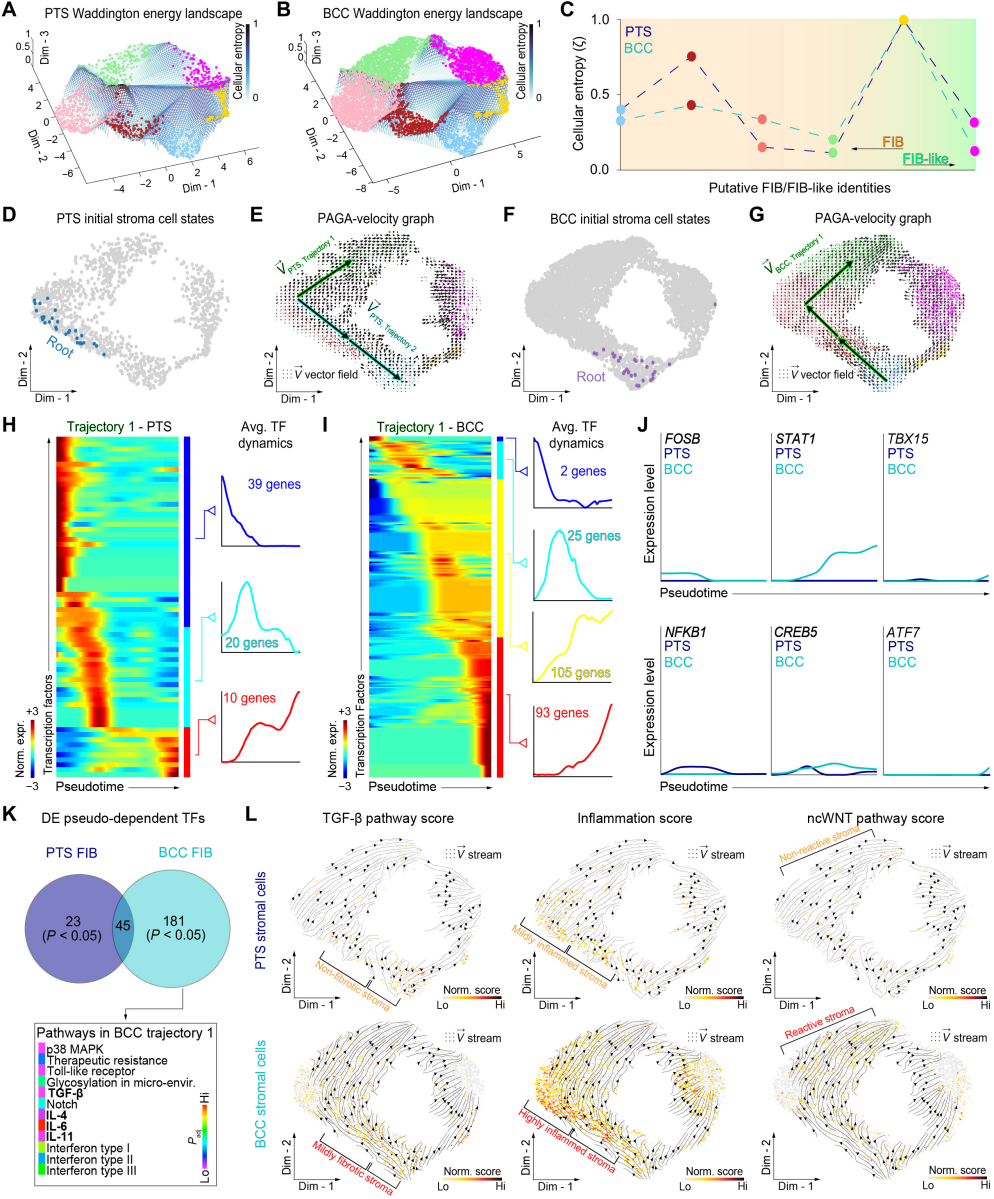
RNA dynamics analyses reveal differential stromal developmental trajectories in human BCC. (**A** and **B**) Three-dimensional Waddington energy (i.e., entropy) landscape of human PTS and BCC. Blue, low entropy; blue, high entropy. (**C**) Quantification of cellular energy. Color of circles corresponds to distinct FIB/FIB-like clusters. Dashed lines connect FIB/FIB-like clusters and are color-coded on the basis of type of condition (i.e., PTS versus BCC). (**D** to **G**) Modeling of initial states in FIB/FIB-like cells suggests distinct developmental trajectories in PTS and BCC stroma. Arrows representing direction of cells’ flow of PAGA-velocity graph were projected as vector field on a two-dimensional embedding. In PTS, bidirectional path of FIBs is represented by trajectory 1 ($\vec{V}$_PTS, Trajectory 1_) and 2 ($\vec{V}$_PTS, Trajectory 2_). In BCC, unidirectional path of FIBs is represented by trajectory 1 ($\vec{V}$_BCC, Trajectory 1_). (**H** and **I**) Rolling-wave plots identify pseudo-dependent TFs overexpressed in PTS (H) and BCC (I) along developmental trajectory 1 and grouped depending on their dynamics (*k* = 3 in PTS; *k* = 4 in BCC). Pseudotime levels are based on normalized counts. Blue, down-regulated TFs; red, up-regulated TFs. (**J**) Comparison of significant pseudo-dependent TFs overexpressed in PTS and BCC developmental trajectories in specific groups. TF dynamics are color-coded on the basis of condition. (**K**) Significant pathway ontologies associated with PTS and BCC FIB developmental trajectory 1 (*P*_adj_ < 0.05). Specific pathway ontologies in BCC are color-coded on the basis of significance. Adjusted *P* value scale shown on the right is based on a rainbow scale. Purple, low significance; red, high significance. (**L**) TGF-β, inflammation, and noncanonical WNT pathway scores based on normalized counts overlaid on two-dimensional embedding with RNA velocity streams reveal specific pathway programs associated with PTS and BCC stromal developmental trajectory 1. Yellow, low score; black, high score.

Our analysis in stroma identified a large degree of cellular FIB and FIB-like heterogeneity in human BCC and PTS at gene expression and regulon levels. To determine whether these cells exist on a continuum or have distinct cellular states, we calculated the cellular entropy (ξ, energy associated with cellular transitions) of BCC and PTS FIB/FIB-like cells using cellular entropy estimator (CEE) (*51*) and visualized their individual CEE scores on three-dimensional Waddington energy landscapes (Fig. 4, A and B). Our results indicated that BCC FIB populations have lower overall entropy than those of PTS and that *TMEM119*^+^ FIBs show the most stability (Fig. 4C), suggesting that FIB I to III may display higher likelihoods of transition to those FIBs that are most juxtaposed to BCC tumor nests (FIB IV). We followed up our analysis with unbiased RNA dynamics–PAGA analyses (*40*, *52*). These complimentary approaches revealed two distinct initial/root states with distinct associated developmental trajectories between BCC and PTS. Focusing on FIBs cells only, PTS FIBs bifurcated toward *WNT5A*^+^ or *ASPN*^+^ termini from a common *CEMIP*^+^ FIB origin (Fig. 4, D and E). In sharp contrast, BCC FIBs followed a unilateral trajectory, emanating mainly from *ASPN*^+^ FIBs and culminating in *TMEM119*^+^ FIBs (Fig. 4, F and G). These observations suggest that a “rewiring” of the tumor stroma may take place to fuel FIBs toward a *TMEM119*^+^/*WNT5A*^+^ state to support tumor growth.

To identify candidate TFs involved in the acquisition of a *TMEM119*^+^/*WNT5A*^+^ state, we extracted FIBs represented in this trajectory, tree-aligned them in pseudotime with Monocle2 (*53*), and performed scEpath analysis (*54*) to identify significant, pseudotime-dependent TFs (α = 0.05). We identified a total of 69 pseudotime-dependent differentially expressed TFs in PTS ($\vec{V}$_PTS, Trajectory 1_) and 225 TFs in BCC ($\vec{V}$_BCC, Trajectory 1_) along trajectory 1 (Fig. 4, H and I). We compared and contrasted TFs from both trajectories by partitioning TFs into groups displaying average TF dynamics, which led to the identification of several genes uniquely present in the BCC trajectory (Fig. 4J). Of interest, *STAT1*, and to a lesser extent *TBX15* and *ATF7*, demonstrated pseudo-dependent expression late in the trajectory in BCC compared to PTS toward *TMEM119*^+^ FIBs. Other TFs displayed early pseudo-dependent trajectories and were shut down in *TMEM119*^+^ FIBs, such as *FOSB* in BCC and *NFKB1* in PTS (Fig. 4J). To gain a broader view of these TFs and identify major pathways in each trajectory compartment, we performed Gene Ontology (GO) analysis on the pseudotime-dependent TFs (Fig. 4K). Among these, we found pathways related to transforming growth factor–β (TGF-β) and inflammation to be significantly expressed. We overlaid these GO terms as a GO biomarker score onto two-dimensional embedding to determine whether their expression was closely associated with the rewiring of tumor stroma and overlaid an RNA velocity stream to visualize and match the movement of the cells with their corresponding GOs (Fig. 4L). *ASPN*^+^ FIBs appear to go through a TGF-β^+^ inflammation state in BCC, but not PTS, before reaching a final reactive stroma status composed of ncWNT signaling–active FIBs—a region high in *WNT5A* ligand. These results suggest that *ASPN*^+^ FIBs are responding to an inflamed microenvironment in BCC and that rewiring of the stroma could arise from inflammatory signals, possibly due to cross-talk with immune cells that have invaded the dermis during BCC progression.

### Inflammatory signaling pathways are active in BCC stroma

**Fig. 5.**
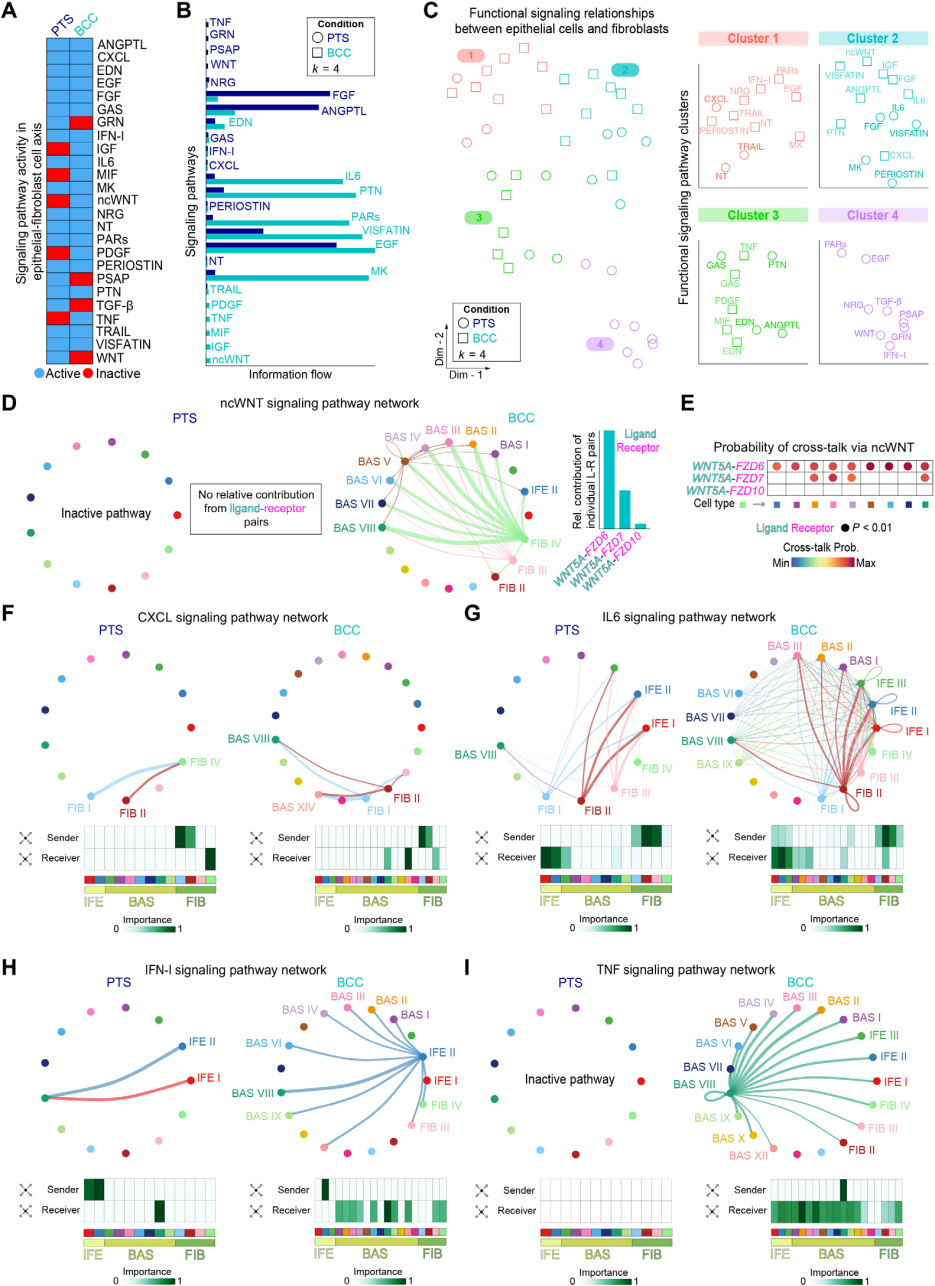
Epithelial-FIB communication modules in human BCC. (**A)** Heatmap of active signaling pathways in epithelial-FIB cross-talk from human PTS and BCC samples. Blue, active signaling pathway; red, inactive signaling pathway. (**B**) Ranking of active signaling pathways in PTS and BCC based on their overall information flow within the inferred cellular networks. Signaling pathways are colored according to condition where they are enriched, whereas those in black are enriched equally across conditions. (**C**) Joint clustering of active signaling pathways from PTS and BCC into a shared two-dimensional manifold according to their functional signaling relationship similarity (*k* = 4). Circles represent PTS signaling pathways; squares represent BCC signaling pathways. Each shape represents the communication network of one signaling pathway. A magnified view of each cluster with labeled active signaling pathways is shown on the right. (**D**) Circle plots show ncWNT signaling in sending and receiving cells. Nodes are colored similarly as senders. Size of cell clusters is representative of the number of active cells in signaling network. ncWNT is active in BCC but not in PTS. Cell types participating in signaling pathway network are labeled. Bar graphs show relative contribution of specific ligand-receptor pairs for ncWNT signaling in BCC. *WNT5A* ligand is the only active ligand in the ncWNT signaling network. (**E**) Dot plots show cross-talk probability between FIBs (senders) and epithelial cells (receivers) via ncWNT signaling. Blue, low cross-talk probability; red, high cross-talk probability. Size of circle represents the percentage of cells with high cross-talk probability. Ligands are colored aqua blue; receptors are colored magenta. (**F** to **I**) Circle plots and network centrality analysis for CXCL (F), IL6 (G), IFN-I (H), and TNF (I) signaling. Only cell clusters participating in signaling network are labeled. Inactive pathway indicates that the pathway is not active. Heatmaps represent network centrality. White, low importance; green, high importance.

How FIB state changes and BCC tumor growth influence each other is unclear. To identify signaling differences between the BCC and PTS microenvironments, we probed the human BCC FIB-epithelial interactome by modeling single cell-cell interactions among *KRT14*^+^ epithelial/tumor and FIB/FIB-like cells using CellChat (*55*). We identified 25 significant signaling pathways active in the stroma-epithelial axis (Fig. 5A). Although most pathways showed signaling activity in both PTS and BCC, GRN (Progranulin), PSAP (Prosaposin), TGF-β, and WNT pathways were inactive in BCC, whereas insulin-like growth factor (IGF), melanocyte release inhibiting factor (MIF), NT (Neurotrophin), PDGF, tumor necrosis factor (TNF), and ncWNT pathways were inactive in PTS (Fig. 5, A and B). The pathways were subdivided into functional signaling relationships between epithelial-derived cells and FIBs, which resulted in four clusters that show similar activities (Fig. 5C). We then identified differentially regulated signaling pathway ligands and receptors between BCC and PTS by comparing the communication probabilities from cell-cell groups. This approach identified ncWNT as a major signaling pathway highly active in BCC compared to PTS (Fig. 5, D and E). The expression of *WNT5A* ligand in PTS FIBs was not significant compared to BCC—with no relative contribution from any ligand-receptor pairs. In sharp contrast, ncWNT signaling was highly active in BCC and mainly driven by *WNT5A* ligand to Frizzled receptors *FZD6*, *FZD7*, and *FZD10*, the latter representing autocrine communication between FIBs (Fig. 5, D and E). A closer look at the probability of cross-talk of *WNT5A* to its receptor shows significant cross-talk to *FZD6* in all the BAS clusters, whereas *FZD7* cross-talk occurs in a subset of BAS clusters (Fig. 5E). In congruence with these observations, we detect significant increases in *WNT5A* expression and a subset of *FZD* receptors in vismodegib-resistant bulk-level RNA-seq data (fig. S9A), and our single-cell expression data show *WNT5A* largely originating from FIB and immune cells and likely interacting with *FZD6* and *FZD7* in the BAS clusters, differentiating epithelia, endothelium, and lymphatic cells (fig. S9, B and C). We also detect tumor-specific in situ expression of *FZD7* adjacent to *WNT5A*^+^ FIBs (fig. S10), suggesting that the cells are in the right location to interact.

WNT5A is a known driver of proinflammatory responses, including CXCL, interferon-I (IFN-I), interleukin 6 (IL6), and TNF (*56*, *57*). CXCL signaling is contained within FIBs in PTS but expands to the BAS cell clusters in BCC (Fig. 5F); IL6 signaling shows greater cross-talk in BCC compared to PTS (Fig. 5G); IFN-I signaling is contained within epithelia in PTS but expands to FIBs in BCC (Fig. 5H); and TNF signaling is exclusive to BCC (Fig. 5I). TNF auto- and paracrine signaling originates from cycling epithelial cells in BCC, signals to other epithelial cells and FIBs, and is an activator of WNT5A (*58*). Together, these results suggest that acute inflammatory signals may be linked to WNT5A activation, which in turn may maintain a proinflammatory state and act as a major inflammatory and stress signaling hub center in BCC stroma.

### Heat shock proteins regulate BCC growth

**Fig. 6.**
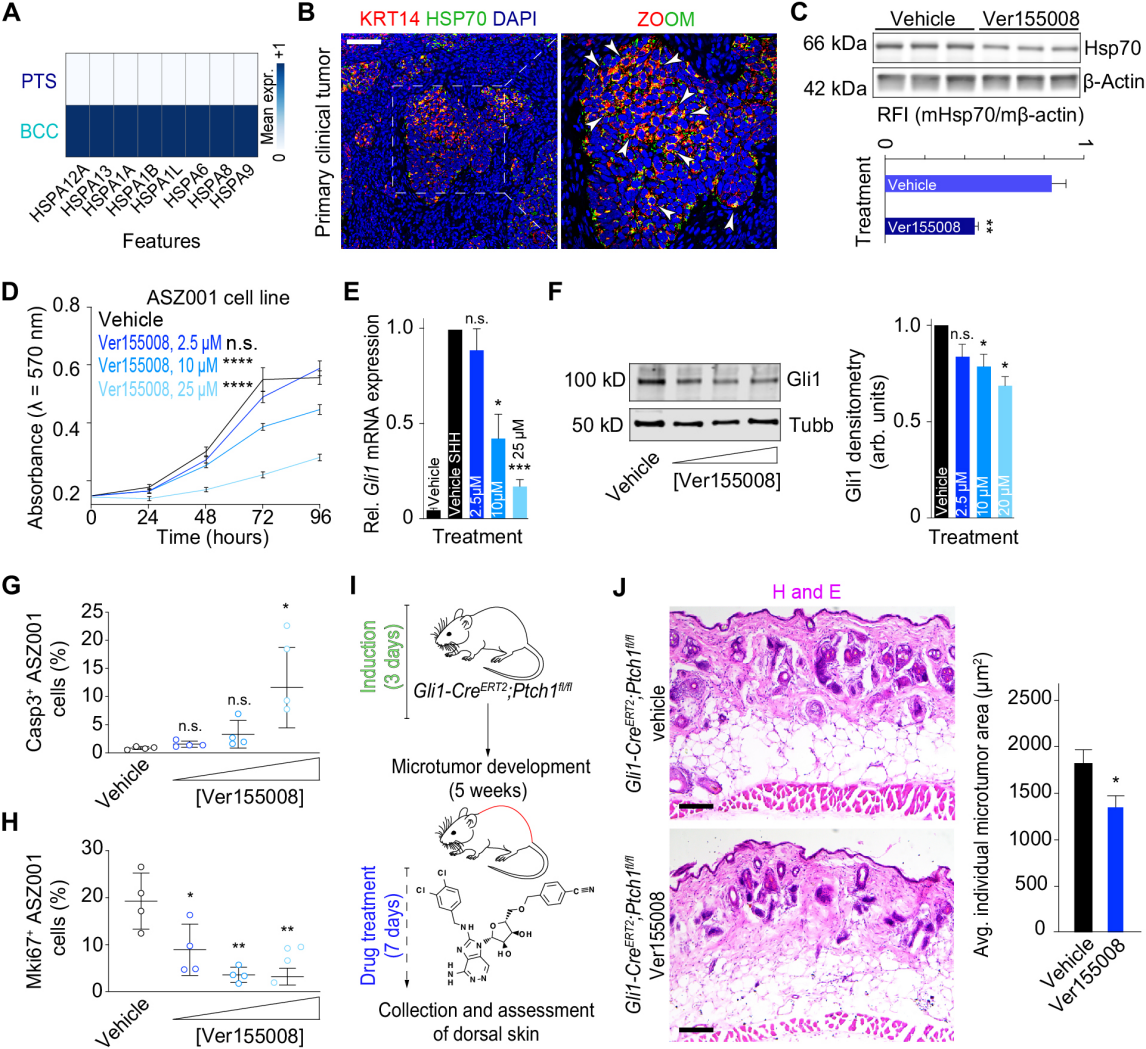
Heat shock proteins are prominent regulators of BCC. (**A**) Heatmap of pseudo-bulk HSP70-coding gene expression in human PTS versus BCC epithelial cells. (**B**) In situ expression of HSP70 protein shows distinct spatial localization in human primary clinical tumors. Inset shows magnified area in BCC nest. White arrows point at HSP70^+^ epithelial cells in BCC nest. Scale bars, 100 μm. (**C**) Western blot and quantification of RFI (Relative Fluorescence Intensity) against Hsp70 in ASZ001 murine cells treated with HSP inhibitor Ver155008. β-Actin served as loading control. Mann-Whitney test (**P = 0.007). (**D** to **F**) HSP inhibitor Ver155008 negatively affects growth of ASZ001 murine cells (D) (two-way ANOVA test; ****P < 0.0001) and down-regulates *Gli1* mRNA (E) and protein expression (F) in vitro in a concentration-dependent manner (unpaired Student’s two-tailed *t* test with Welch’s correction; *P < 0.05 and ***P < 0.001). Tubb served as loading control. Experiments were repeated at least three times, and data are represented as the means ± SEM. (**G** and **H**) HSP inhibitor Ver155008 significantly induces apoptosis via Casp3 (H) and negatively affects proliferation (G) via Mki67 in ASZ001 murine cells in vitro in a concentration-dependent manner. Bar graphs represent the mean of nine replicate wells ± SEM. Unpaired Student’s two-tailed *t* test (*P < 0.05 and **P < 0.01). (**I**) Schematic representation of microtumor development and HSP inhibitor treatment in *Gli1-Cre^ERT2^;Ptch1^fl/fl^* mice. Ver155008- and vehicle-treated dorsal skin tissues were collected and assessed for microtumors. (**J**) H&E of Ver155008- and vehicle-treated *Gli1-Cre^ERT2^;Ptch1^fl/fl^* mouse dorsal skin tissues. Scale bars, 100 μm. Quantification of microtumor surface area in vehicle- and Ver155008-treated *Gli1-Cre^ERT2^;Ptch1^fl/fl^* mouse dorsal skin tissues. Bars represent average individual microtumor area ± SEM. Surface area decreased in a concentration-dependent manner compared to vehicle-treated control mice. Unpaired Student’s two-tailed *t* test (*P < 0.05).

Our BCC FIB-epithelial interactome modeling revealed an inflammatory and stress signaling hub in BCC stroma. To determine how a proinflammatory response from FIBs may influence tumor growth and progression, we looked for relevant differentially expressed genes in BCC versus PTS epithelia and found HSP genes up-regulated in BCC compared to PTS epithelia (Fig. 6A). Their expression levels are largely not significant in bulk-level RNA-seq of vismodegib-resistant BCC compared to normal skin (fig. S11, A and B), reinforcing the advantage of gene expression at single-cell resolution. HSPs are an adaptive response to cellular stress and inflammation and have been strongly implicated in cancer development and progression (*59*). We identified eight HSP70-coding family genes that were significantly up-regulated in BCC compared to PTS epithelial cells, including *HSP12A2*, *HSPA13*, *HSPA1A*, *HSPA1B*, *HSPA1L*, *HSPA6*, *HSPA8*, and *HSPA9* (Fig. 6A and fig. S11, A and B). We then spatially resolved the expression of HSP70 in situ using protein immunostaining coupled with high-resolution confocal imaging. We found that KRT14^+^ BCC nests expressed cytoplasmic HSP70, with seldom interfollicular epithelial epithelial cells and nonepithelial cells expressing the protein (Fig. 6B and fig. S11, C and D). A primary infiltrative BCC with perineural invasion demonstrated nuclear expression of HSP70 (fig. S11D).

To determine whether HSPs are important for BCC cell growth, we used the HSP70 inhibitor Ver155008 on the vismodegib-resistant murine BCC cell line ASZ001 (*12*, *60*) and observed decreased protein expression of Hsp70 and a dosage-dependent inhibition of ASZ001 cell proliferation (Fig. 6, C and D). Two other HSP family inhibitors, KNK437 (pan-HSP inhibitor that includes HSP70) and ganetespib (HSP90 inhibitor), also showed a dosage-dependent inhibition of ASZ001 cell proliferation (fig. S11, E and F). HSP90 genes are also significantly enriched in our single-cell data, but not in bulk-level RNA-seq (fig. S10, A and B), suggesting that some BCC tumor cells may use HSPs as a general mechanism to promote tumor cell growth. Ver155008 treatment resulted in a decrease in *Gli1* expression, a downstream HH target gene, at both RNA and protein levels (Fig. 6, E and F), suggesting that HSP70 may be a novel HH pathway regulator. HSP70 inhibition affected both proliferation and survival of the vismodegib-resistant BCC cells as determined by Mki67 and Casp3 staining quantification (Fig. 6, G and H). Last, we aimed to determine the role of HSPs on BCCs in vivo using the BCC mouse model *Gli1-Cre^ERT2^;Ptch1^fl/fl^* (*61*). We induced *Gli1-Cre^ERT2^;Ptch1^fl/fl^* mice with tamoxifen for three consecutive days to generate BCC microtumors, followed by intraperitoneal injection with vehicle control or Ver155008 daily for 7 days (Fig. 6I). Histological staining of the dorsal skin of Ver155008-treated mice showed significant reduction in microtumor area compared to vehicle-treated controls (Fig. 6J). Our in vitro and in vivo studies help to reconcile our scRNA-seq analysis and identify HSPs, particularly HSP70 family members, as potential new regulators of BCC tumor growth and HH signaling and may offer a novel therapeutic venue for the treatment of BCCs.

## DISCUSSION

Functional heterogeneity in human BCC has largely been explored using bulk-level genomic and transcriptomic studies where it was difficult to separate out distinct cell types and clonality within tumors and their unique contributions to BCC pathogenesis (*28*, *62*, *63*). Using single-cell technologies, we identified the milieu of cell types and states that make up BCC and found that bulk-level studies can provide complementary datasets but often lead to identification of significant genes that are nonspecific and broadly expressed across different cell types due to the heterogeneity of normal and cancerous cells in biopsy samples (figs. S6 and S7). When analyzed at the single-cell level, we found additional BCC biomarkers that better define BCCs and label tumors from specific donors that further highlight the heterogeneity of this disease. We also identified spatial heterogeneity in FIBs that led to an oncogenic trajectory favoring *TMEM119*^+^/*WNT5A*^+^ reactive stroma and inflammatory signals that create a burst of cell-cell cross-talk between FIBs and BCC epithelial cell clusters. Last, our results suggest that BCC tumors may respond to inflammatory signals from the stroma by expressing HSPs and that HSP inhibitors may serve as an effective therapeutic strategy to suppress HH signaling and tumor growth.

Our efforts to distinguish between malignant and normal cells between and within biopsy samples to create a more nuanced BCC gene signature highlighted the importance of integration benchmarking. Although no algorithm is unflawed (*64*), we demonstrated that use of benchmark integration using several different methods increases user confidence in clustering of the underlying data. BCCs are highly heterogeneous and have the highest mutation frequency out of all cancers (*62*), making integration of multiple samples difficult. All five clustering algorithms we used (Seurat, SCTransform, LIGER, Harmony, and scMC) showed remarkable efficiency in correctly clustering nonepithelial cell types with low mutational burden (Fig. 1, C to E, and fig. S4), but epithelial cells with higher mutational burden showed significant batch effects in clustering. In our experience, Seurat, SCTransform, LIGER, and Harmony could not distinguish between normal and malignant cells, often separating donor samples from each other regardless of origin. However, scMC—and pySCENIC via regulon activity (fig. S5, D and E) (*34*)—clustered normal and malignant epithelial cells distinctly while maintaining cohesion within each condition (Fig. 1D), likely due to its ability to learn a shared reduced dimensional embedding of cells to retain biological variation while removing technical variation associated with each sample (*26*).

Despite the difficulty in integrating epithelial cells, stromal cell states displayed remarkable cohesion between PTS and BCC samples. Four FIB states and two FIB-like states were found in both normal and malignant samples, suggesting that CAFs may be an active state of normal tissue-resident FIBs and that cancer-specific stromal states do not occur in BCC. However, there is a large degree of active remodeling that occurs in BCC stroma, likely driven mainly by collagen and metalloproteinase gene products (Fig. 3I). Furthermore, joined RNA velocity–PAGA analysis suggests that highly inflamed stroma expressing TGF-β and IL genes, classic activators of CAFs (*65*), give rise to reactive stroma highlighted by *WNT5A*^+^ FIBs (Fig. 4L). This cancer-specific rewiring of the stroma goes from an ASPN^+^ state (FIB I) to a CLIC2^+^ (FIB II) and CEMIP^+^ (FIB III) state found sparingly around KRT14^+^ tumor nests, before reaching the *TMEM119*^+^/*WNT5A*^+^ state (FIB IV) that surrounds KRT14^+^ tumor nests at a relatively high density compared to the other three FIB states (Fig. 3, E to H and M). *TGFB1* and general inflammatory genes are expressed throughout the first three FIB populations and may provide a mechanism of activation to the *WNT5A*^+^ state, while WNT5A may reinforce this signaling as it is a known driver of proinflammatory signals to induce an immune response (*56*, *57*). Stromal rewiring driven by inflammation and CAFs are promising therapeutic targets (*65*), and our GRN analysis suggests that the JAK-STAT pathway may regulate *WNT5A* expression (Fig. 5, N and O), opening up the possibility for JAK-STAT inhibitors in treating BCC patients (*66*).

How CAFs and general FIB inflammation affect BCC tumor growth is unclear. Our CellChat inferred signaling results suggest a burst of signaling between FIBs and BAS clusters involving CXCL, IL6, IFN-1, and TNF pathways (Fig. 5, F to I). WNT5A is a known driver of each of these pathways (*56*, *57*), and TGF-β1 and inflammatory signals like IL6 and TNF are known activators of CAFs and WNT5A in particular (*65*). With this influx of inflammatory signals, BCCs may respond by up-regulating HSPs as a protection mechanism (*59*), although this mechanism may be indirect given that WNT5A treatment in combination with HH ligand does not significantly affect HSP70 protein levels (fig. 11G). HSPs are known to have significant roles in DNA repair mechanisms to maintain genome stability and integrity, a process that is heavily intertwined with inflammation (*67*). Cancers live on a “double-edged sword” where they need enough genomic instability to thrive, but not too much instability to adversely alter successful replication (*68*). Cancer-specific HSP expression may help maintain the genomic instability balance to promote tumor growth, which may explain our results that show that HSP inhibitors are effective at suppressing BCC growth. Although HSP inhibitors, especially HSP90 inhibitors, have general cytotoxicity issues as all tissues require continuous molecular chaperone activity to ensure proper folding of essential proteins (*69*), HSP70 inhibitors may provide a useful alternative route to therapy as Hsp70 knockout mice are healthy (*70*), the protein is dispensable for growth of nontransformed cells (*71*), and HSP70 inhibition shows distinct effects compared to HSP90 inhibitors (*72*). Our data suggest that short-term HSP70 inhibitor treatment may be better tolerated systemically in the *Gli1-Cre^ERT2^;Ptch1^fl/fl^* murine model (Fig. 6J), and BCCs have the advantage of topical treatment that may allow better toleration to toxic compounds (*73*). In addition, HSP inhibition may be more effective with combinatorial treatment, a likely future direction, as evidenced by ongoing clinical trials in several cancer types (*74*).

Overall, our findings illustrate the heterogeneity and dynamic nature of the BCC cellular ecosystem. The signaling relationships between BCC epithelial cells and FIBs revealed a WNT5A-mediated inflammatory signature that led to the discovery of an HSP-specific protective mechanism that is necessary to maintain tumor growth. Further characterizing these types of responses may provide additional mechanistic insight into the complicated cross-talk between the tumor and its microenvironment and provide additional avenues for therapeutic suppression of skin cancer.

## MATERIALS AND METHODS

### Ethics statements

Human clinical studies were approved by the Ethics Committee and Institutional Review Board of Stanford University Hospital (Palo Alto, California, USA). We certify that all applicable institutional regulations concerning the ethical use of information and samples from human volunteers were strictly followed in this work. Each subject provided written informed consent. All animal studies were performed in strict adherence to the Institutional Animal Care and Use Committee (IACUC) guidelines of the University of California, Irvine (AUP-21-006).

### Human samples

A total of six surgically discarded human tissues (BCC, *n* = 4; PTS, *n* = 2) were obtained from excisional biopsy specimens at Stanford University Hospital (Palo Alto, California, USA). BCCs were classified into superficial, nodular, and infiltrative BCC (ID: BCC-I); superficial and nodular BCC (ID: BCC-II); unknown/“hybrid” (ID: BCC-III); and infiltrative with perineural invasion BCC (ID: BCC-IV) subtypes by a board-certified dermatopathologist. All data collection and anonymous analysis were approved by the Institutional Review Board of Stanford University Hospital.

### Mice

The following mice were used in this study: *Gli1-Cre^ERT2^* (JAX #007913) and *Ptch1^fl/fl^* (JAX #012457) (*61*). *Gli1-Cre^ERT2^;Ptch1^fl/fl^* mice were genotyped by polymerase chain reaction (PCR). Briefly, genomic DNA was collected from mouse toes and lysed in DirectPCR lysis reagent as per the manufacturer’s protocol (Fisher Scientific). Genomic DNA was amplified using Taq polymerase (Apex), and products were resolved on a 2% agarose gel (Apex). The following primers were used: CreER, 3′-CATGCTTCATCGTCGGTCC-5′ (forward) and 3′-GATCATCAGCTACACCAGAG-5′ (reverse); Ptch1, 3′-AGTGCGTGACACAGATCAGC-5′ (forward) and 3′-CCCAATTACCCATCCTTCCT-5′ (reverse).

### Microtumor induction and drug treatment

Microtumors were induced in the skin of 6-week-old *Gli1-Cre^ERT2^;Ptch1^fl/fl^* mice (of indiscriminate gender), by administering 100 μl of tamoxifen (10 mg/ml; Sigma-Aldrich) intraperitoneally for three consecutive days. Five weeks later, mice were treated with either dimethyl sulfoxide (DMSO; vehicle) or Ver155008 (16 mg/kg) intraperitoneally for seven consecutive days. The final volume of all injections was 100 μl. At the end of treatment, mice were sacrificed, and dorsal skin were collected, fixed in 4% paraformaldehyde, immersed in 30% sucrose, and frozen in Tissue-Tek OCT (Optimal Cutting Temperature) compound (Sakura, Japan). Samples were cryosectioned at 14 μm. Unless otherwise noted, at least five mice were used for each treatment condition.

### Microtumor assessment

Frozen mouse dorsal skin tissues were cryosectioned at 14 μm and stained with hematoxylin and eosin (H&E) (Thermo Fisher Scientific). Images were taken at ×200 magnification on an AmScope microscope with an AmScope MU500B digital camera. Microtumor size was assessed as the sum of total microtumor area and as average size per microtumor and quantified using FIJI software (*75*). Statistical analysis was performed using GraphPad Prism, and application of Student’s two-tailed *t* test was used to dictate statistical significance (*P < 0.05).

### Histology and immunohistochemistry

Discarded human tumor skin tissues were processed at the Department of Pathology at Stanford University and sectioned at a thickness of 5 μm. Immunostaining was performed on paraffin sections. Heat-based antigen retrieval was performed when necessary. Tissue sections were blocked in either 3% bovine serum albumin (BSA) or 3% donkey serum. The following primary antibodies were used: rabbit anti-CLIC2 (Abcam; 1:50), rabbit anti-CEMIP (Proteintech; 1:100), rabbit anti-TMEM119 (Proteintech; 1:50), chicken anti-KRT14 (BioLegend; 1:1000), mouse anti-LHX2 (Santa Cruz Biotechnology; 1:50), rabbit anti-ACTA2 (Abcam; 1:250), mouse anti-KRT15 (Santa Cruz Biotechnology; 1:50), rabbit anti-HSP70 (Proteintech; 1:100), rabbit anti-CASP3 (R&D Systems; 1:1000), and rabbit anti-MKI67 (Abcam; 1:1000). Secondary chicken (Abcam), rabbit (Life Technologies), and mouse (Life Technologies) were used at a concentration of 1:1000. Sections were counterstained with 4′,6-diamidino-2-phenylindole (DAPI) (Vector Laboratories). Images were acquired on an Olympus FV3000 confocal laser scanning microscope.

#### Histology and hematoxylin and eosin staining

OCT-embedded skin tissues of DMSO vehicle control or Ver155008-treated mice were cryosectioned at a thickness of 10 μm using the CryoStar NX50 cryostat (Thermo Fisher Scientific). Cryosections were incubated at 55°C for 10 min, washed in phosphate-buffered saline (PBS) before performing a standardized H&E staining protocol using Gill’s 3 formulation Hematoxylin (Thermo Fisher Scientific) and Eosin Y at pH 4.7 (Thermo Fisher Scientific) for counterstaining and clearing of the stain with xylene-based solutions, and mounted with a toluene-based mounting medium (Permount, Fisher Scientific). Images were acquired with the 10× objective on an AmScope bright-field microscope with a MU500B digital camera.

#### RNA in situ hybridization

Frozen tissue sections were processed for RNA in situ hybridization using the RNAscope Multiplex Fluorescent Detection Kit v2 [323100, Advanced Cell Diagnostics (ACD)] as per the manufacturer’s protocol. The following ACD probes were used in this study: Human (Hs): *ASPN* (402731-C2), *KRT14* (813871), *WNT5A* (316791-C2), and *FZD7* (414061-C4). Slides were counterstained with DAPI (Vector Laboratories). Images were acquired on an Olympus FV3000 confocal laser scanning microscope.

#### Cell culture and growth assay

Vismodegib-resistant murine BCC ASZ001 cells were grown in 154CF medium containing chelated 2% fetal bovine serum (FBS; Life Technologies), 1% penicillin-streptomycin (Life Technologies), and 0.07 mM CaCl_2_ (Life Technologies). NIH 3T3 cells were grown in Dulbecco’s modified Eagle’s medium (Life Technologies) containing 10% FBS (Life Technologies) and 1% penicillin-streptomycin (Life Technologies) and were incubated in a water-jacketed incubator at 37°C with 5% CO_2_ output. Cells were seeded at a density of 1000 cells per well into 96-well flat-bottom plates. After 24 hours, cells were treated with DMSO (vehicle control) or varying concentrations of Ver155008 (MedChemExpress), KNK437 (Thermo Fisher Scientific), or ganetespib (Thermo Fisher Scientific) consecutively for 2, 4, and 6 days. Growth assay was performed with MTT 3-(4,5-(dimethylthiazol-2-yl)-2,5-diphenyl tetrazolium bromide) (Sigma-Aldrich) as per the manufacturer’s protocol. Proliferation (MKI67) and apoptosis (CASP3) were determined by immunostaining fixed cells at the indicated time points. Unless otherwise noted, experiments were repeated at least three times, and data are represented as the mean of nine replicate wells ± SEM. Statistical analysis was performed using GraphPad Prism, and application of Student’s two-tailed *t* test and two-way analysis of variance (ANOVA) test was used to dictate significance (*P < 0.05, **P < 0.01, and ***P < 0.001; n.s., not significant).

#### Cell culture and treatments

NIH 3T3 cells were seeded to confluence, serum-starved, or serum-starved in 1:100 SHH-N conditioned medium with DMSO (vehicle control) or Ver155008 (MedChemExpress) at various concentrations for 24 hours. RNA was isolated using the Direct-zol RNA MiniPrep Plus (ZYMO Research). Quantitative reverse transcription PCR was performed using the iTaq Univer SYBR Green 1-Step Kit (Bio-Rad) on the StepOnePlus Real-time PCR System (Applied Biosystems). The fold change in mRNA expression of the HH target gene *Gli1* was measured using ΔΔ*C*_t_ analysis with *Gapdh* as an internal control gene. The following primers were used: *Gli1*, 5′-GCAGGTGTGAGGCCAGGTAGTGACGATG-3′ (forward) and 5′-CGCGGGCAGCACTGAGGACTTGTC-3′ (reverse); *Gapdh,* 5′-AATGAATACGGCTACAGCAACAGGGTG-3′ (forward) and 5′-AATTGTGAGGGAGATGCTCAGTGTTGGG (reverse). Vismodegib-resistant murine BCC ASZ001 cells were grown in 154CF/PRF (Thermo Fisher Scientific) medium containing 2% FBS (Life Technologies/GIBCO; 10437028), both heat-inactivated and chelated, 0.07 mM CaCl_2_, and 1% penicillin-streptomycin (Life Technologies/GIBCO) until confluent in six-well plates. Cells were then treated with either SHH-N conditioned medium or SHH-N conditioned medium in combination with Wnt5a recombinant protein (200 ng/ml; R&D Systems) for 24 hours or with single treatment of vehicle control (DMSO) or Ver155008 (25 μM) inhibitor for 24 hours. Unless otherwise noted, experiments were repeated at least three times, and data are represented as the mean of triplicates ± SEM. Statistical analysis was performed using GraphPad Prism, and application of Student’s two-tailed *t* test was used to dictate statistical significance (*P < 0.05, **P < 0.01, and ***P < 0.001).

#### Protein immunoblotting

Protein extraction was performed in control- and 24-hour–treated vismodegib-resistant murine BCC ASZ001 cells grown in six-well plates using 250 μl of 2× SDS loading buffer (100 mM tris-HCl, 1 M dithiothreitol, 4% SDS, and 0.2% bromophenol blue) and by shaking for 30 min at 250 rpm at 4°C. Denatured proteins (10 μl) were ran in a Mini-PROTEAN TGX precasted SDS-PAGE gel (Bio-Rad) with 4 to 20% polyacrylamide gradient and bis-acrylamide cross-linker and blotted onto a nitrocellulose membrane (0.45 μm; Prometheus). Membranes were probed with primary antibodies [1:1000 in 5% skimmed milk in TBST (tris-buffered saline and Tween 20)] overnight at 4°C. The following primary antibodies were used: rabbit anti-Hsp70 (Proteintech), mouse anti–β-actin (BioTechne), and mouse β-tubulin [E7, DSHB (Developmental Studies Hybridoma Bank)]. Fluorescence bands were visualized using an Odyssey CLx Li-Cor imaging system by incubating for 1 hour at room temperature with conjugated secondary antibodies. The following secondary conjugated antibodies were used: donkey anti-rabbit Alexa Fluor 680 (1:5000 in 5% skimmed milk in TBST; The Jackson Laboratory) or donkey anti-mouse Alexa Fluor 790 (1:5000 in 5% skimmed milk in TBST; The Jackson Laboratory). The relative fluorescence intensity of proteins of interest was quantified using FIJI software (*75*) and normalized to a housekeeping protein (β-actin or β-tubulin). Statistical analysis was performed using GraphPad Prism, and application of Mann-Whitney test was used to dictate statistical significance (*P < 0.05 and **P < 0.01).

#### Cell isolation and 3′-droplet–enabled scRNA-seq

Adjacent peritumor and tumor skin specimens were surgically excised from human donors at Stanford University Hospital (Palo Alto, CA, USA) and immediately shipped to University of California, Irvine (Irvine, CA, USA). Within 24 hours, excised tissues were minced and incubated in a dispase II (Sigma-Aldrich) and collagenase IV (Sigma-Aldrich) solution overnight at 4°C. Cells were incubated in 0.25% trypsin-EDTA for 15 min at 37°C and quenched with chelated FBS. Cells were passed through a 40-μm filter and centrifuged at 1500 rpm for 5 min, and the pellet was resuspended in keratinocyte serum-free medium supplemented with Epidermal Growth Factor 1-53 and Bovine Pituitary Extract (Life Technologies; 17005042). After isolation, cells were resuspended in PBS free of Ca_2_^+^ and Mg_2_^+^ and 1% BSA and stained with SYTOX Blue Dead Cell Stain (Thermo Fisher Scientific). Samples were bulk-sorted at 4°C on a BD FACSAria Fusion using a 100-μm nozzle (20 PSI) at a flow rate of 2.0 with a maximum threshold of 3000 events per second. After exclusion of debris and singlet/doublet discrimination, cells were gated on viability. Live cells were resuspended in 0.04% UltraPure BSA (Sigma-Aldrich) and counted using the automated cell counter Countess (Thermo Fisher Scientific). Cells were captured using Chromium (10X Genomics). GEM (Gel Bead-In EMulsions) generation, barcoding, post–GEM-RT (reverse transcription) cleanup, complementary DNA (cDNA) amplification, and cDNA library construction were performed using Single-Cell 3′ v2 chemistry (10X Genomics). cDNA libraries were sequenced on an Illumina HiSeq4000 platform (Illumina) [one lane, 100 PE (Paired End)]. Cell counting, suspension, GEM generation, barcoding, post–GEM-RT cleanup, cDNA amplification, library preparation, quality control, and sequencing were performed at the Genomics High Throughput Sequencing facility at the University of California, Irvine.

#### 3′-Droplet–enabled scRNA-seq raw data processing

Transcripts were aligned to the human reference genome (GRCH38/transcriptome) using Cell Ranger (version 2.1.0). Sequencing metrics for each library are as follows: (PTS-I) Sequencing metrics: ~264,949,873 total number of reads and ~98.7% valid barcodes; mapping metrics: ~93.1% reads mapped to genome, ~91.0% reads mapped confidently to genome, and ~71.1% reads mapped confidently to transcriptome; cell metrics: ~7164 estimated number of cells, ~92.9% fraction reads in cells, ~36,983 mean reads per cell, ~2382 median genes per cell, ~21,853 total genes detected, ~9238 median UMI (unique molecular identifier) counts per cell. (PTS-II) Sequencing metrics: ~317,022,706 total number of reads and ~98.7% valid barcodes; mapping metrics: ~93.1% reads mapped to genome, ~91.0% reads mapped confidently to genome, and ~71.1% reads mapped confidently to transcriptome; cell metrics: ~7164 estimated number of cells, ~92.9% fraction reads in cells, ~36,983 mean reads per cell, ~2382 median genes per cell, ~21,853 total genes detected, and ~9238 median UMI counts per cell. (BCC-I) Sequencing metrics: ~170,434,662 total number of reads and ~98.5% valid barcodes; mapping metrics: ~91.3% reads mapped to genome, ~89.0% reads mapped confidently to genome, and ~67.6% reads mapped confidently to transcriptome; cell metrics: ~10,025 estimated number of cells, ~90.2% fraction reads in cells, ~17,000 mean reads per cell, ~2484 median genes per cell, ~22,986 total genes detected, ~6618 median UMI counts per cell. (BCC-II) Sequencing metrics: ~128,178,058 total number of reads and ~98.5% valid barcodes; mapping metrics: ~92.8% reads mapped to genome, ~90.6% reads mapped confidently to genome, and ~70.4% reads mapped confidently to transcriptome; cell metrics: ~12,487 estimated number of cells, ~86.8% fraction reads in cells, ~10,264 mean reads per cell, ~1708 median genes per cell, ~22,737 total genes detected, and ~4361 median UMI counts per cell. (BCC-III) Sequencing metrics: ~335,812,707 total number of reads and ~98.3% valid barcodes; mapping metrics: ~87.5% reads mapped to genome, ~84.8% reads mapped confidently to genome, and ~65.7% reads mapped confidently to transcriptome; cell metrics: ~7094 estimated number of cells, ~82.6% fraction reads in cells, ~47,337 mean reads per cell, ~2315 median genes per cell, ~23,364 total genes detected, and ~8292 median UMI counts per cell. (BCC-IV) Sequencing metrics: ~277,281,459 total number of reads and ~98.6% valid barcodes; mapping metrics: ~93.2% reads mapped to genome, ~90.6% reads mapped confidently to genome, and ~62.6% reads mapped confidently to transcriptome; cell metrics: ~8829 estimated number of cells, ~88.8% fraction reads in cells, ~31,405 mean reads per cell, ~1983 median genes per cell, ~23,362 total genes detected, and ~5516 median UMI counts per cell.

#### Doublet/multiplet simulation and low-quality cell pruning

Putative doublets/multiplets were simulated with Single-Cell Remover of Doublets (Scrublet) (version 0.2.1) (*76*) using raw count matrices. The number of neighbors used to construct the KNN (K Nearest Neighbors) classifier of observed transcriptomes and simulated doublets/multiplets was set as default. The doublet/multiplet score threshold was adjusted manually as suggested by the developer. Briefly, digital matrices for putative singlets were used for low-quality cell pruning using a user-defined pipeline. Viable singlets were kept and used for downstream query and comparative analyses if and only if they met the following collective quality control criteria: (i) 350 < genes/cell < 5000; (ii) cells contained no more than 10% of mitochondrial gene expression; (iii) cells were not identified as outliers (*P* = 1 × 10^−3^) (*77*).

#### Data processing and benchmarking of 3′-droplet–enabled scRNA-seq

##### Processing of individual datasets

Preprocessed digital matrices from individual tumor datasets were processed using Seurat (version 4.0.1). Seurat objects were created and log-normalized with a scale factor of 10,000. Variable features were identified using vst with top 2000 features. Data were scaled, and metadata variables, including mitochondrial gene expression, were regressed. Principal components analysis was calculated using variable features identified using a combination of heuristic and statistical approaches. Individual datasets were visualized using a two-dimensional embedding.

##### Benchmarking of integrated datasets

Individual datasets from PTS and BCC were processed for integration, downstream analyses, or visualization with Seurat (version 3.0.0900) (*20*), Single-Cell Transform (version 0.3.2) (*23*), LIGER (version 2.0.1) (*78*), Harmony (version 0.1.0) (*25*), or scMC (version 1.0.0) (*26*) as suggested by each developer with minor modifications to source code. Of note, cells from PTS scoring high for appendage-related genes (*79*) were excluded from integration and anchoring, as well as from downstream query and comparative analyses.

#### Statistical analyses

Statistical analysis was performed using GraphPad Prism Software (v5.02). Differences between groups were assessed using unpaired Student’s two-tailed *t* test, unpaired Student’s two-tailed *t* test with Welch’s correction, two-way ANOVA test, or Mann-Whitney test. A *P* value smaller than 0.05 was considered statistically significant.

## Supplementary Materials

### This PDF file includes:

Supplementary experimental procedures and methods Figs. S1 to S11 Tables S1 to S3 References

## Notes

View/request a protocol for this paper from *Bio-protocol*.
