# Supplementary material for: Single-cell analysis of human basal cell carcinoma reveals novel regulators of tumor growth and the tumor microenvironment
Source: Sci Adv. 2022 Jun 10;8(23):eabm7981. doi: 10.1126/sciadv.abm7981 (PMC9187229; doi:10.1126/sciadv.abm7981)
Supplement: Supplementary file 1 — Supplementary experimental procedures and methods Figs. S1 to S11 Tables S1 to S3 References [file sciadv.abm7981_sm.pdf]

Science Advances
AAAS

Supplementary Materials for

Single-cell analysis of human basal cell carcinoma reveals novel regulators of tumor growth and the tumor microenvironment

Christian F. Guerrero-Juarez *et al.*

Corresponding author: Scott X. Atwood, satwood@uci.edu; Kavita Sarin, ksarin@stanford.edu

Sci. Adv. **8**, eabm7981 (2022)
DOI: 10.1126/sciadv.abm7981

This PDF file includes:

Supplementary experimental procedures and methods
Figs. S1 to S11
Tables S1 to S3
References

Supplementary experimental procedures and methods

Copy number variation analyses. To infer genomic copy number structure, we performed InferCNV (version 1.7.1) as per developer's suggestions using standard parameters (inferCNV of the Trinity CTAT Project. https://github.com/broadinstitute/inferCNV). We used a cutoff of 0.1 for the minimum average read counts per gene among reference cells. We used non-immune, non-epithelial, and non-appendage epithelial cells as internal reference control.

Gene marker analyses. To identify shared (i.e., general) and unique (i.e., subtype/donor-specific) BCC gene signature profiles in our dataset, we performed differential gene expression analysis (i.e., gene marker identification) using Seurat (version 4.0.1). For this, we implemented a minimum average Log_2 fold change in expression (0.25x) and a minimum percent of cells that must express gene marker in either one cluster (0.50 or 50%). Gene markers were identified using the *FindAllMarkers* function using the Wilcoxon Rank Sum test.

Marker gene module scoring. Aggregate marker gene module scores were assigned to early epithelial differentiation, late epithelial differentiation, and BCC identity score using the AddModuleScore function in Seurat with the following conditions enabled: ctrl = 2.5 or 5. Basal, non-differentiating and basal late differentiating epithelial cells were identified by implementation of a "Early differentiation" and "Late differentiation" signatures defined by a core set of known markers as previously described (41). BCC cells were identified by implementation of a BCC signature defined by a core set of known markers as previously described (27). HH- and WNT-active and responsive cells were identified by implementation of a "Hedgehog signaling aggregate score" or "WNT signaling aggregate score" defined by a core set of known HH and WNT ligands and receptors, respectively as previously described in GSEA (80). Scored HH and WNT-active/responsive cells were colored distinctly. Double-positive cells were color-coded based on a blend threshold score scale. Aggregate marker gene module and blend threshold scores were Log-normalized and visualized in two-dimensional feature plots.

Quantification of single cells. To quantify cells expressing our genes of interest, we instituted the following logical statements: *Gene A*$^+$ (i.e., *BCAM*) represents a group of cells whose gene expression for "Gene A" per cell in a group (i.e., condition) exceeds the maximum expression for "Gene A" among all cells multiplied by a constant of 0.8. Such notation is also applied to other genes of interest (i.e., *EPCAM*, *TP63*, *LGALS1*, *MYLK*, *CALML5*, *SCGB2A2*, or *KRT19*). If a cell belongs to the cluster *BCAM*$^+$, *EPCAM*$^+$, *TP63*$^+$, and *LGALS1*$^+$, then that particular cell will be

clustered as “Quadrupled^{+}”. For all the other cases, the cell is clustered as “None”. Calculations were carried out in MATLAB (Version 9.5).

Visualization of cell density. Cell density plots were calculated using Nebulosa (version 1.0.2) and overlaid on a two-dimensional embedding (81).

Differential gene expression analyses. Differentially expressed genes between PTS and BCC fibroblasts were calculated using DESeq2 as previously described and with minor modifications tailored for the analysis of scRNA-seq data sets (49). Hypothesis testing was performed with the Wald test and differentially expressed genes for a particular comparison (i.e., PTS versus BCC from anyone cluster) were filtered using a P-adjusted threshold of 0.05 and a fold-change of 1.5x in either direction.

Gene ontology analyses. Gene Ontology (GO) analysis was performed with Panther (82), DAVID (83), and Enrichr (84, 85). GOs were visualized by heatmap.

RNA dynamics analyses. RNA dynamics in single cells was calculated using scVelo (40). First, we generated loom files using the Python script *velocyto.py* (Python version 2.7.2) for each individual library. Loom files for individual libraries from a particular condition, PTS I-II or BCC I-IV, were combined using loompy (version 2.0.16). Velocity vectors were estimated using scVelo (version 0.2.2) based on the dynamic model of RNA velocity with default parameters. Velocity vectors were overlaid on a two-dimensional embedding. Predicted root and terminal states were based on Markovnikov reaction diffusion. Initial states were also calculated with CellRank (52). Root/initial and terminal states were visualized on a two-dimensional embedding.

Pseudotime analyses. Pseudo-ordering of individual fibroblasts (FIBs) from peri-tumor skin (PTS) or basal cell carcinoma (BCC) was performed using Monocle2 (Version 2.10.1) (53, 86). Briefly, FIB cells from PTS or BCC trajectory 1 were subclustered and a *cellDataSet* object was created in Monocle2 with the function *newCellDataSet* with standard arguments enabled. Subclustered FIBs were ordered based on variable features and DDRTree-based dimensionality reduction was performed using the *reduceDimensions* function. To identify differentially expressed pseudotime-dependent transcription factor changes, we applied single cell Energy path (scEpath; Version 1; MATLAB Version 9.5) (54) on Monocle2-ordered PTS or BCC FIBs. Statistically significant pseudotime-dependent gene changes were identified by comparing the

standard deviation of the observed smoothed expressions with a set of similarly permuted expressions by randomly permuting the cell order (*nboot* = 100 permutations). We considered all genes with a standard deviation greater than 0.01 and a Bonferroni-corrected P-value below a significance level α = 0.05 to be pseudotime-dependent. Human transcription factors were identified using the Animal Transcription Factor Database (AnimalTFDB 2.0) (87) by enabling the *TF_Ifo.human.Symbol* function. Pseudotime-dependent genes were represented and visualized using a rolling wave plot with user-defined optimal K-means clustering.

Cellular entropy estimation. Cellular entropy estimation was performed as previously described with minor modifications and projected on a three-dimensional Waddington energy landscape (51).

Cell-cell communication analyses. Cell-cell communication networks were modeled based on abundance of ligand-receptor pair transcripts with CellChat (version 0.5.0) (88). To infer cell-cell communication network differences and similarities between PTS and BCC cells, we sub-clustered epithelial and fibroblasts from individual conditions. Cell groups of interest were merged, normalized and used as input for CellChat. We calculated over-expressed genes and the significant ligand-receptor interactions with the *identifyOverExpressedGenes* (thresh.p = 0.05) and *identifyOverExpressedInteractions* functions, respectively. We used the human database of ligand-receptor pairs provided by CellChat. The communication probabilities were calculated with *computeCommunProb* (tresh = 0.05, nboot = 100, Hill function parameter kn = 0.5) and inferred the cellular communication network at a signaling pathway level using *computeCommunProbPathway* with default parameters (tresh = 0.05). We filtered out cell-cell communications where a minimum of 5 cells per group were present. The aggregated cell-cell communication networks were calculated with *aggregateNet* (tresh = 0.05) with default parameters. To identify conserved and induced/perturbed communication networks in PTS vs. BCC epithelial-fibroblast cells, we lifted cells from PTS condition and performed joint manifold and classification learning analyses.

Gene Regulatory Network analyses. Gene Regulatory Networks were modeled with pySCENIC (34, 89) (version 0.10.2) in a Python Environment (version 3.7). Briefly, we used a pre-defined list of human transcription factors (TFs) (https://github.com/aertslab/pySCENIC/blob/master/resources/hs_hgnc_tfs.txt) and inferred regulatory interactions between them and their putative target genes with GRNBoost2. We

focused on activating modules only and used them for downstream query and analyses. We performed cisTarget motif enrichment based on a ranking and recovery approach with a 10kb putative regulatory region boundary from the TSS (hg38__refseq-r80__10kb_up_and_down_tss.mc9nr.feather) (https://resources.aertslab.org/cistarget/). AUC was used to assess gene recovery and regulon activity and also used for dimensionality reduction. The activity of regulons across epithelial and fibroblast/fibroblast-like cells were compared against each other and across conditions by converting Regulon Specificity Scores (RSS) to Z-scores as previously described. Regulon-specific modules were identified from the network inference output using iRegulon (http://iregulon.aertslab.org). Regulon targets were correlated with differentially expressed genes in specific clusters across conditions.

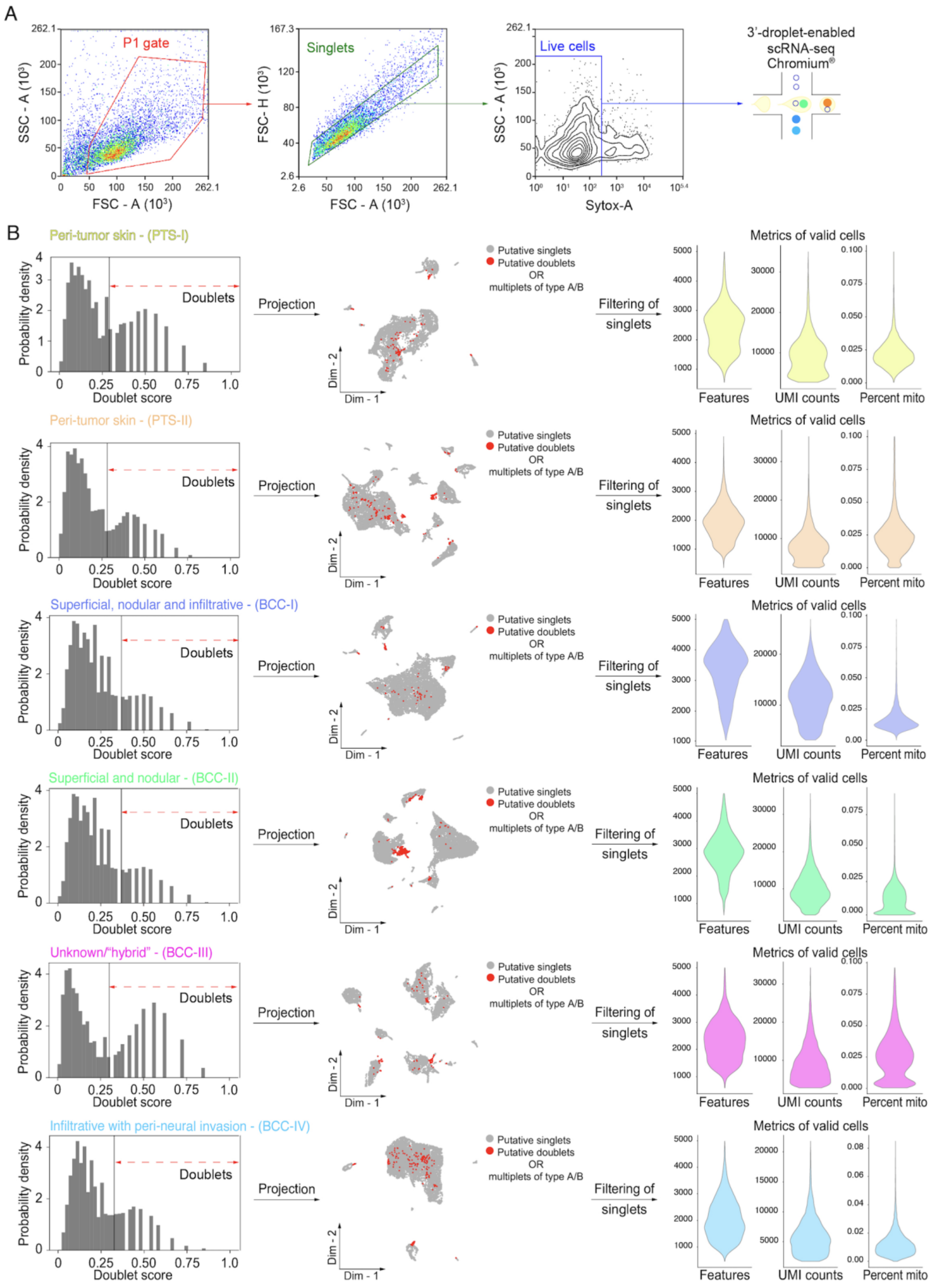
A
P1 gate
SSC - A (10³)
FSC - A (10³)
Singlets
FSC- H (10³)
FSC - A (10³)
Live cells
SSC - A (10³)
Sytox-A
3'-droplet-enabled
scRNA-seq
Chromium®
B
Peri-tumor skin - (PTS-I)
Peri-tumor skin - (PTS-II)
Superficial, nodular and infiltrative - (BCC-I)
Superficial and nodular - (BCC-II)
Unknown/"hybrid" - (BCC-III)
Infiltrative with peri-neural invasion - (BCC-IV)
Probability density
Doublet score
Doublets
Projection
Dim - 1
Dim - 2
Putative singlets
Putative doublets
OR
multiplets of type A/B
Filtering of
singlets
Metrics of valid cells
Features
UMI counts
Percent mito

Supplementary Figure 1. Cell sorting strategy and quality control filtering of single cells. A. Schematic representation of fluorescent activated cell sorting (FACS) and downstream 3'-droplet-enabled single-cell RNA-sequencing of epithelial and stromal cells from human PTS and BCC primary clinical tumors. **B.** Bimodal distribution of predicted singlet and doublet/multiplet PTS (PTS-I and PTS-II) and BCC – superficial, nodular, and infiltrative (BCC-I); superficial and nodular (BCC-II); unknown/"hybrid" (BCC-III); and infiltrative with perineural invasion (BCC-IV) cells. Straight, solid line indicates user-defined doublet score threshold. Putative singlets and doublets were projected onto a two-dimensional embedding and labeled accordingly. Gray – putative singlets; red – putative doublets/multiplets of type A and B. Putative singlets were processed for low-quality cell pruning and removal. On the right, metrics showing distribution of features/cell, UMI counts/cell, and percentage of mitochondrial genes/cell in valid cells post-cell pruning and filtering are visualized as violin plots and color coded accordingly. Valid cells were used in downstream query and comparative bioinformatic and computational analysis.

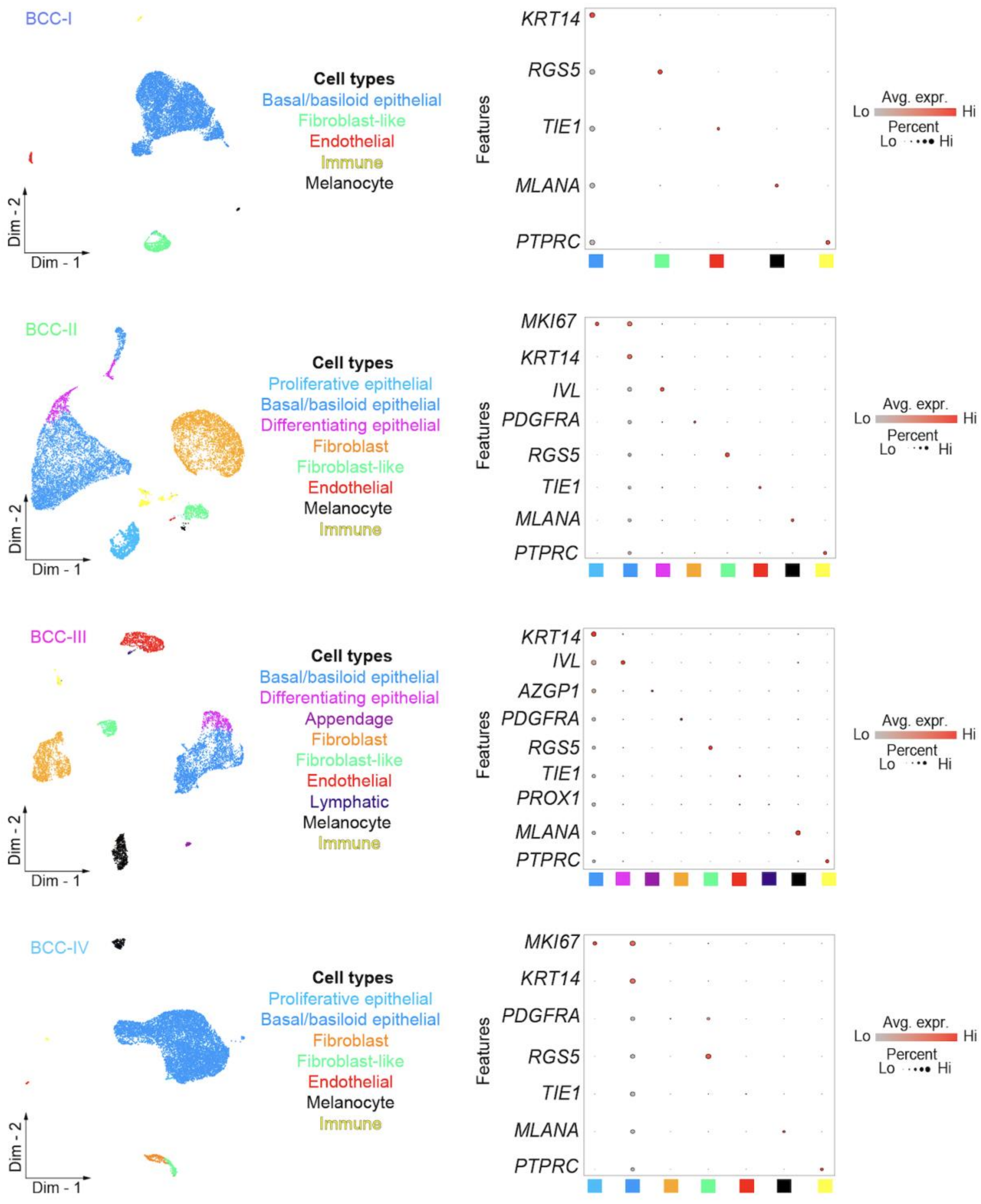

Supplementary Figure 2. Cell type identification and characterization in basal cell carcinoma subtypes. Two-dimensional clustering of single cells isolated from individual human

BCC subtypes reveals cellular heterogeneity in BCCs. IDs represent subtype and donor and are color-coded accordingly. BCC subtypes include: superficial, nodular, and infiltrative (BCC-I); superficial and nodular (BCC-II); unknown/"hybrid" (BCC-III); and infiltrative with perineural invasion (BCC-IV). Ten total distinct meta-clusters are identified at various proportions across BCC subtypes and labeled on the right. Bona fide marker for each cell type is visualized using dot plots. Gray – low average gene expression of canonical and marker genes; red – high average gene expression of canonical and marker genes. Size of circle represents the percentage of cells expressing canonical and marker genes.

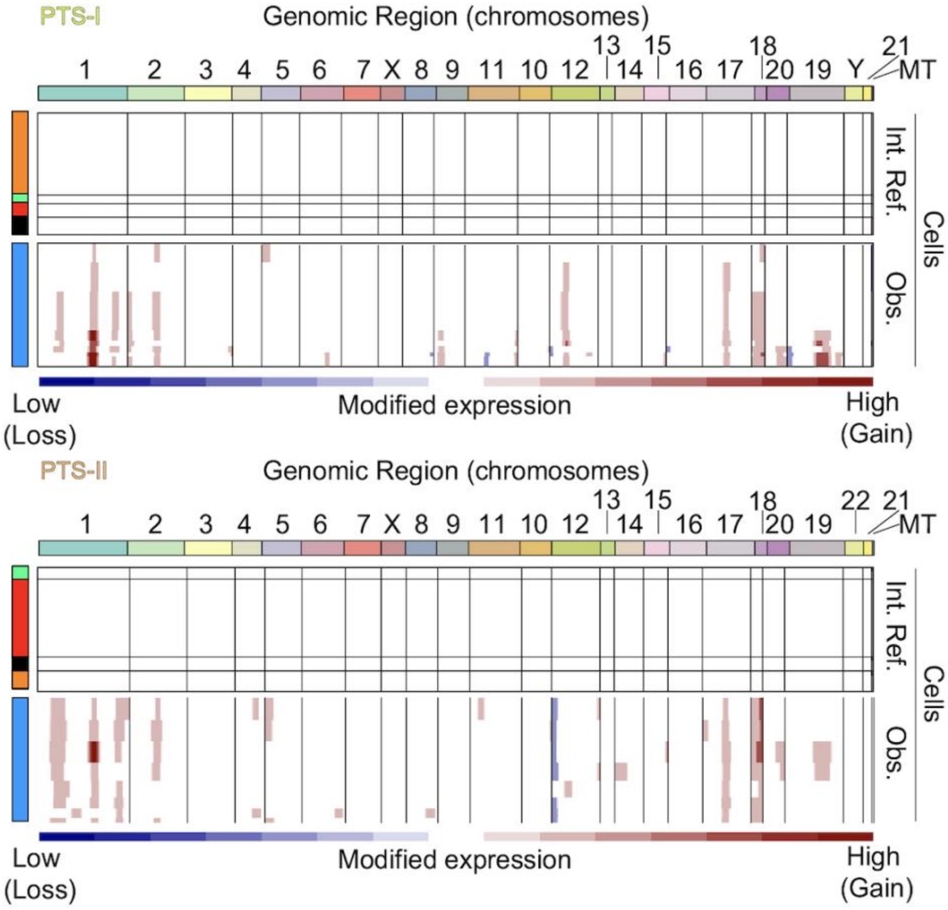

Supplementary Figure 3. Copy number variant analysis in peri-tumor skin and basal cell carcinoma subtypes. Copy number variant analysis of epithelial cells in human peri-tumor skin (PTS-I and PTS-II) with InferCNV. Blue represents low modified expression – corresponding to genomic loss; red represents high modified gene expression – corresponding to genomic gain. Internal reference (Int. Ref) cells refer to non-epithelial, non-immune control cells. Observations (Obs.) refer to putative malignant epithelial cells. Genomic regions (chromosomes) are labeled and color-coded.

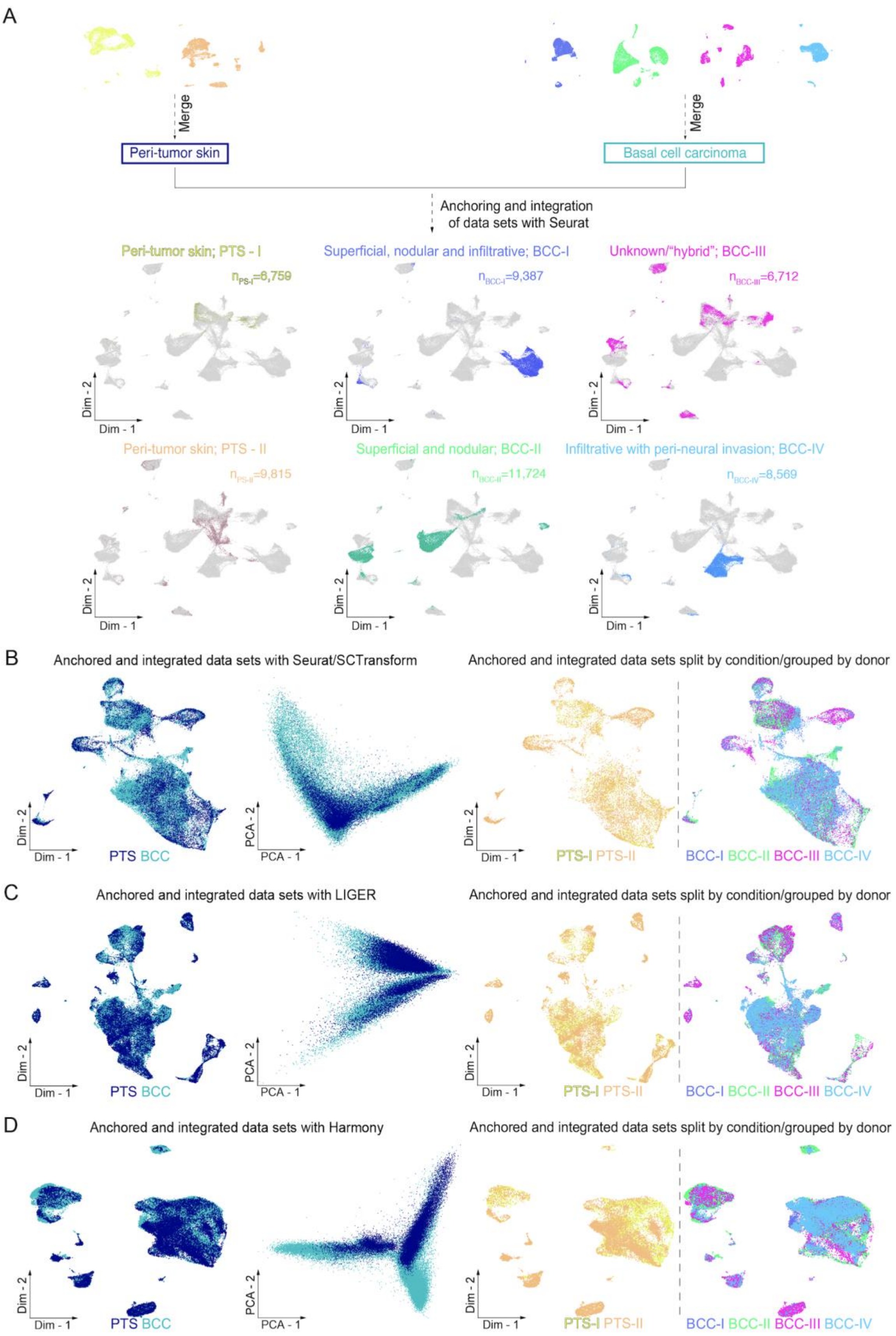
A
Merge
Peri-tumor skin
Merge
Basal cell carcinoma
Anchoring and integration
of data sets with Seurat
Peri-tumor skin; PTS - I
n$_{PS-I}$=6,759
Superficial, nodular and infiltrative; BCC-I
n$_{BCC-I}$=9,387
Unknown/"hybrid"; BCC-III
n$_{BCC-III}$=6,712
Peri-tumor skin; PTS - II
n$_{PS-II}$=9,815
Superficial and nodular; BCC-II
n$_{BCC-II}$=11,724
Infiltrative with peri-neural invasion; BCC-IV
n$_{BCC-IV}$=8,569
Dim - 2
Dim - 1
B
Anchored and integrated data sets with Seurat/SCTransform
Anchored and integrated data sets split by condition/grouped by donor
PTS BCC
PCA - 2
PCA - 1
PTS-I PTS-II
BCC-I BCC-II BCC-III BCC-IV
C
Anchored and integrated data sets with LIGER
Anchored and integrated data sets split by condition/grouped by donor
D
Anchored and integrated data sets with Harmony
Anchored and integrated data sets split by condition/grouped by donor

Supplementary Figure 4. Benchmarking integration of peri-tumor skin and basal cell carcinoma subtypes. A-C. Clustering of human PTS and BCC data sets with Seurat (**A**), SCTransform (**B**), LIGER (**C**), or Harmony (**D**). Clustering was visualized with two distinct, two-dimensional embeddings. PTS and BCC data sets are color coded and split by condition and grouped by donor.

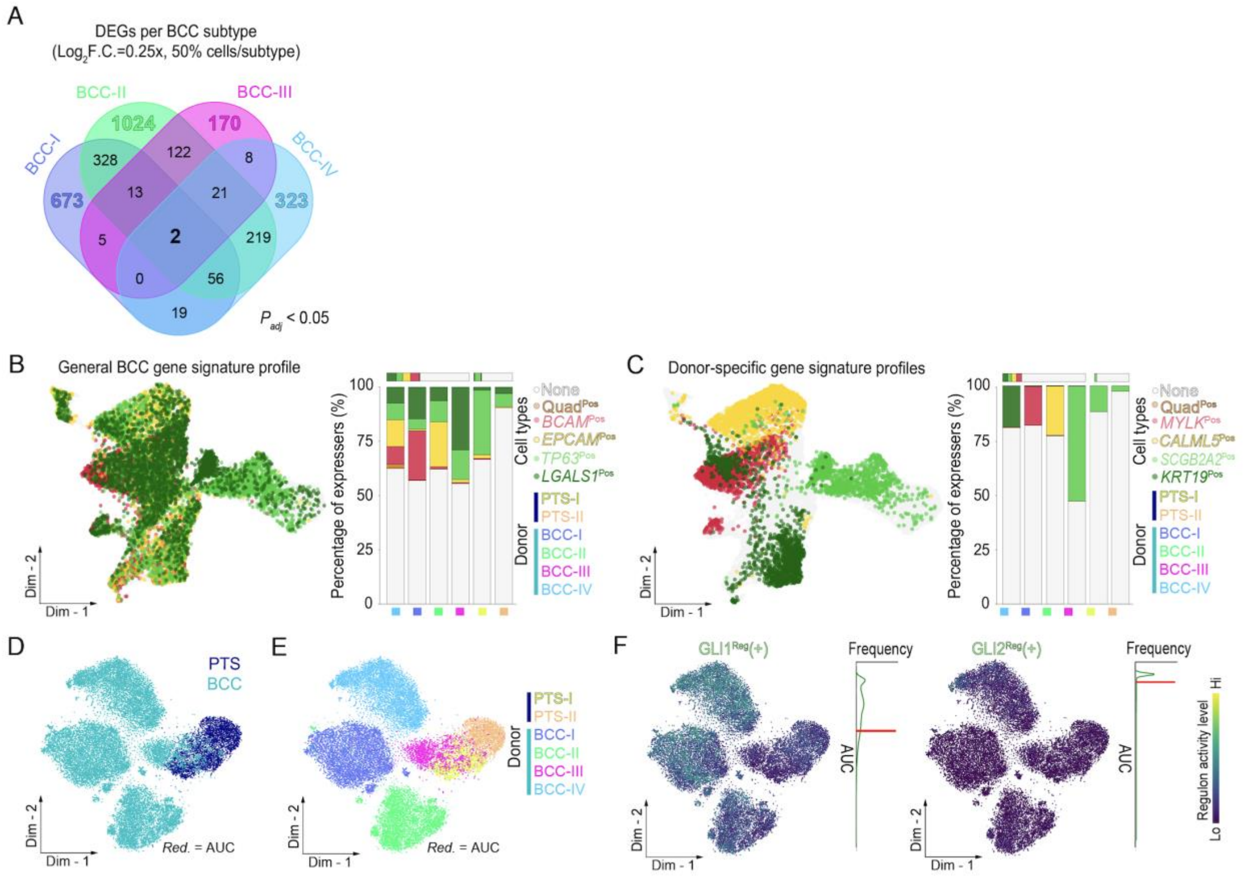

Supplementary Figure 5. Comparative analysis in peri-tumor skin and basal cell carcinoma subtypes. A. Four-way Venn diagram showing differentially expressed genes (DEGs) in BCC-I, BCC-II, BCC-III, and BCC-IV with respect to each other. DEGs per BCC subtype are considered unique and significant if 50% of cells in each condition express the gene at a Log_2 F.C. of 0.25x ($P_{adj} < 0.05$; Wilcoxon Rank Sum test). **B, C.** General BCC signature from this data overlaid on a two-dimensional epithelial cell embedding. Percentage of cells expressing *BCAM*, *EPCAM*, *TP63*, and *LGALS1*. Quadrupled positive cells are labeled orange and negative cells are labeled gray. Donor-specific gene signature from this data overlaid on two-dimensional epithelial cell embedding. Percentage of cells expressing *BCAM*, *EPCAM*, *TP63*, and *LGALS1*. Quadrupled positive cells are labeled orange and negative cells are labeled gray. **D, E.** Regulon activity was used for dimensionality reduction and regulons for PTS and BCC epithelial cells were visualized in a two-dimensional embedding and colored by tissue type and donor. **F.** pySCENIC-predicted $GLI1^{Reg}$(+) and $GLI2^{Reg}$(+) regulons for BCCs. Regulon activity was used for dimensionality reduction and visualized in a two-dimensional embedding. Density plots represent AUC distribution per regulon selected. Purple – low regulon activity; yellow – high regulon activity.

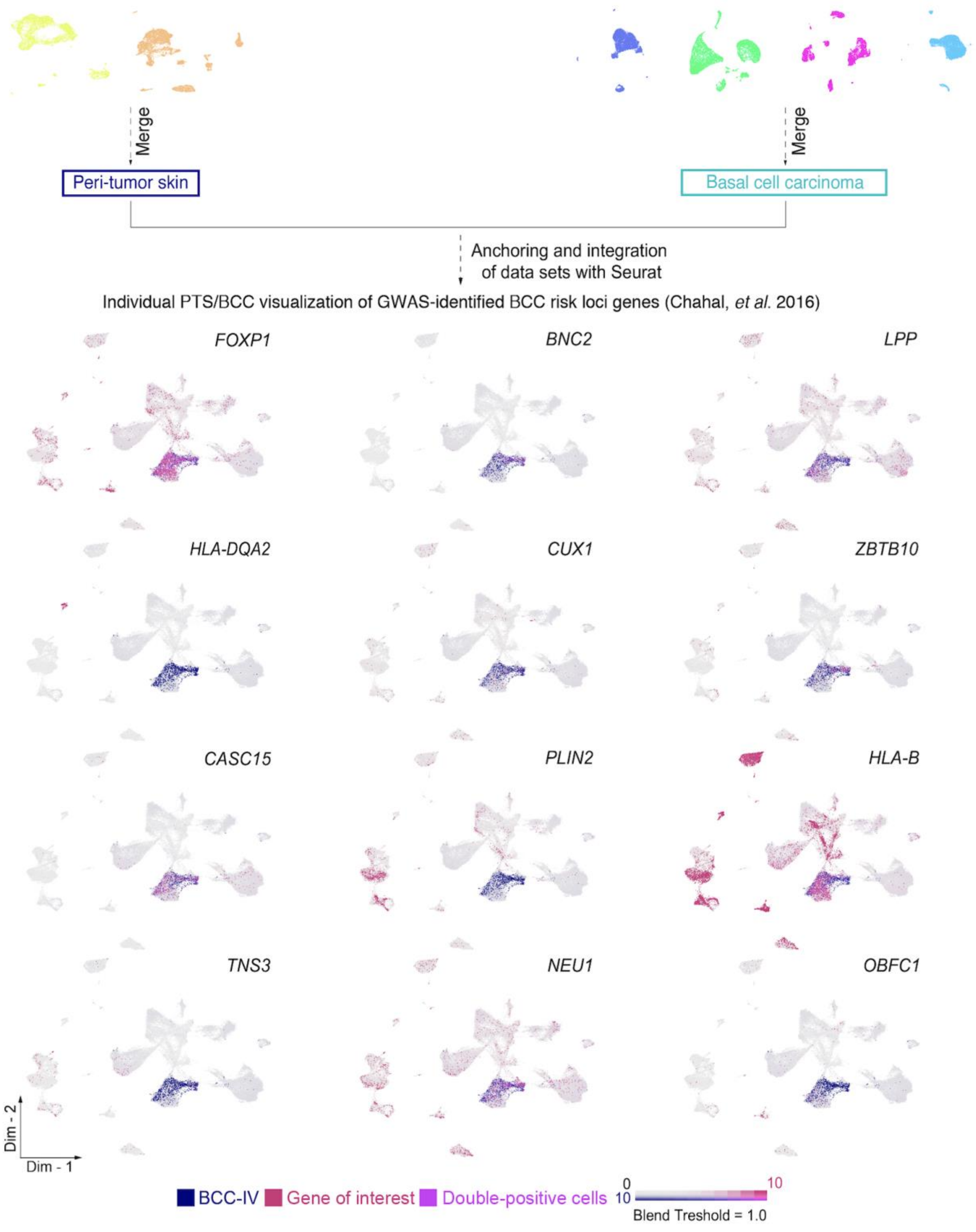

Supplementary Figure 6. Projection of genes identified from basal cell carcinoma-risk loci GWAS analyses. Integration of human PTS and BCC data sets with Seurat for visualization

purposes. Feature plots show expression of GWAS-identified BCC risk loci genes in Chahal *et al*. *(39).* Gray – no expression; BCC-IV identity was colored blue. Genes (i.e., gene loci) of interest were colored magenta. Double-positive cells were color-coded based on a blend threshold score (blend threshold = 1.0). Aggregate marker gene module and blend threshold scores were Log-normalized and visualized in two-dimensional feature plots.

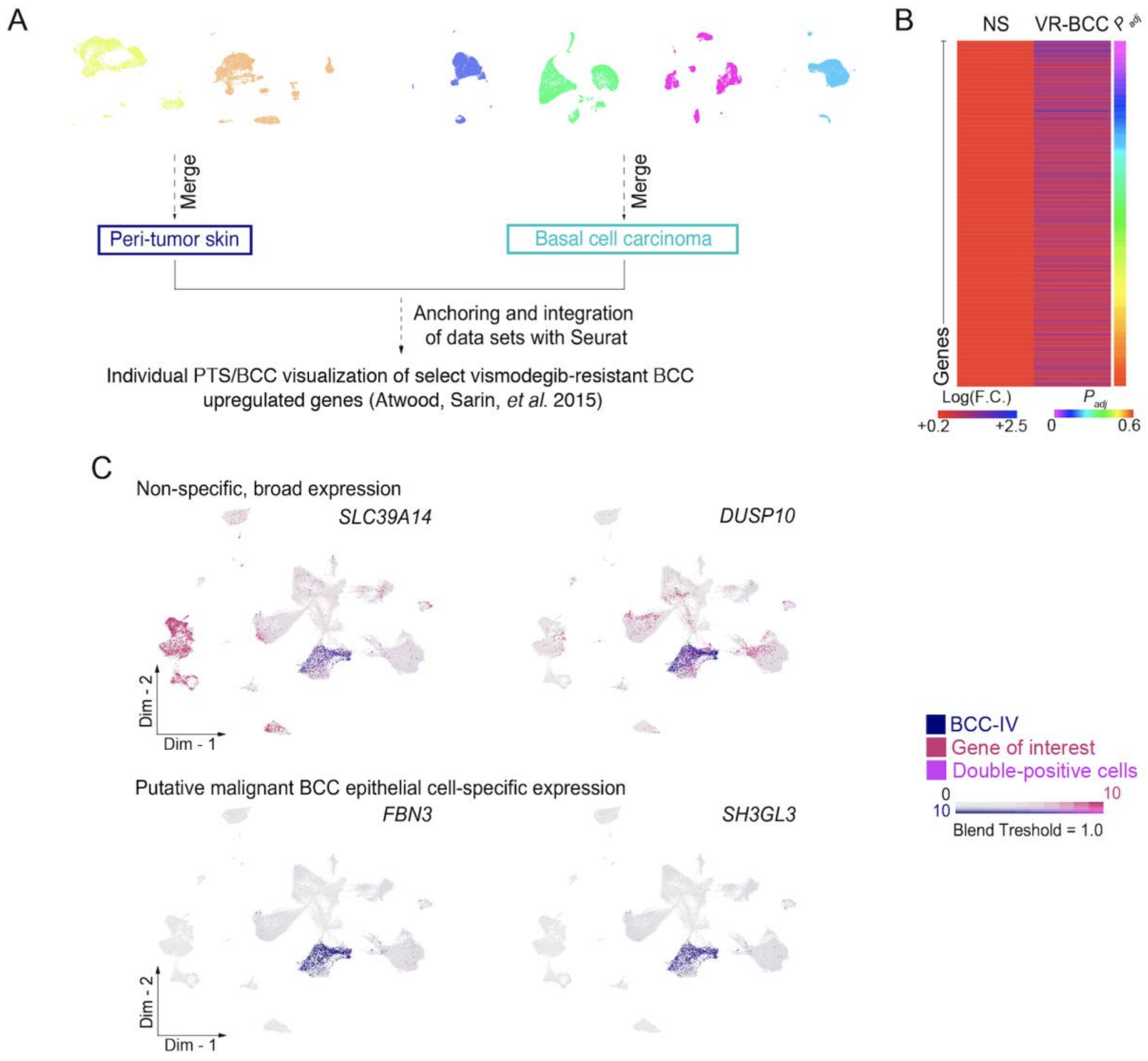

Supplementary Figure 7. Projection of genes identified from comparative bulk-level RNA-sequencing studies. **A.** Integration of human PTS and BCC data sets with Seurat for visualization purposes. **B.** Bulk-level RNA-sequencing analysis. Heatmap of differentially expressed genes in normal skin vs. vismodegib-resistant BCCs identified in Atwood *et al*. (*28*). Red – no change; blue – upregulation based on a Log fold change scale. Adjusted P-value scale is shown on the right based on a rainbow scale. Purple – most significant; red – least significant. Genes of interest are on shown on the right. **C.** Feature plots show expression of indicated marker genes categorized as "non-specific, broad expression" and "putative malignant BCC epithelial cell-specific expression". Gray – no expression; BCC-IV identity was colored blue. Genes of interest were colored magenta. Double-positive cells were color-coded based on a blend

threshold score (blend threshold = 1.0). Aggregate marker gene module and blend threshold scores were Log-normalized and visualized in two-dimensional feature plots.

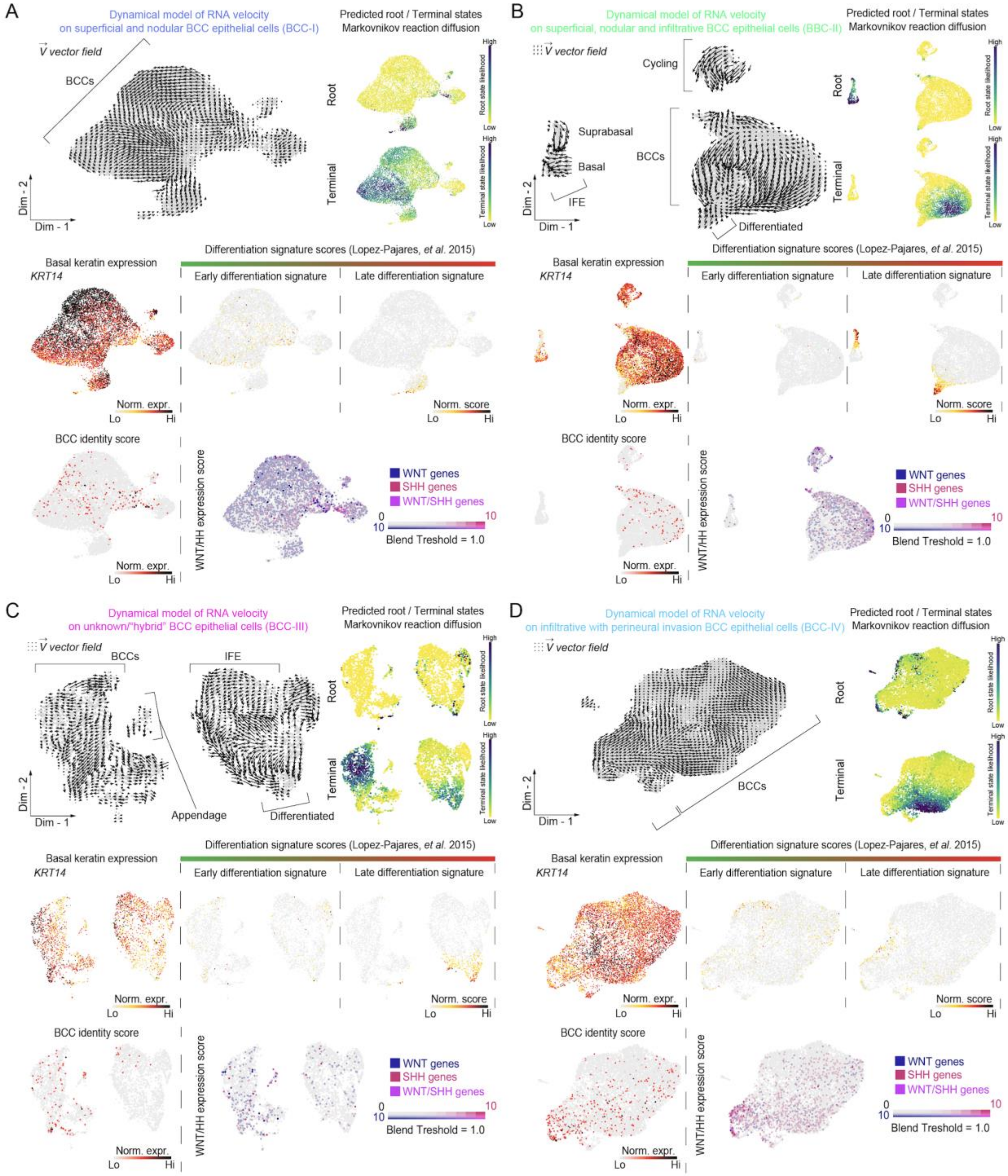

Supplementary Figure 8. Intra-tumoral RNA dynamics analysis of individual basal cell carcinoma subtypes. A-D. Two-dimensional sub-clustering of epithelial cells isolated from individual human BCC subtypes based on basal *KRT14* expression. BCC subtypes include: (**A**) superficial, nodular, and infiltrative (BCC-I); (**B**) superficial and nodular (BCC-II); (**C**) unknown/“hybrid” (BCC-III); and (**D**) infiltrative with perineural invasion (BCC-IV). RNA velocity analysis reveals distinct intra-tumoral dynamics in BCC epithelial cells. RNA velocity was computed based on spliced/unspliced ratios as described in Bergen *et al*. (*40*). Resultant arrows were projected as vector field on a two-dimensional embedding. Arrows represent direction of cells’ flow. Intra-tumoral predicted initial/root and terminal states based on Markovnikov reaction diffusion are presented for each BCC subtype. Yellow – low probability; purple – high probability for root and terminal states. Differentiation signature scores depicting early, and late epidermal differentiation are presented for each BCC subtype. Gray – low normalized gene expression based on normalized counts; black – high normalized gene expression based on normalized counts. BCC identity score projected on two-dimensional embedding. Gray – low normalized gene expression based on normalized counts; black – high normalized gene expression based on normalized counts. Hedhehog (HH) and WNT-active/responsive cells were colored distinctly. Double-positive cells were color-coded based on a blend threshold score (blend threshold = 1.0). Aggregate marker gene module and blend threshold scores were Log-normalized and visualized in two-dimensional feature plots. Gray – no expression; blue – cells which scored high for WNT-related genes; magenta – cells which scored high for HH-related genes; and purple – cells which scored high for both WNT- and HH-related genes.

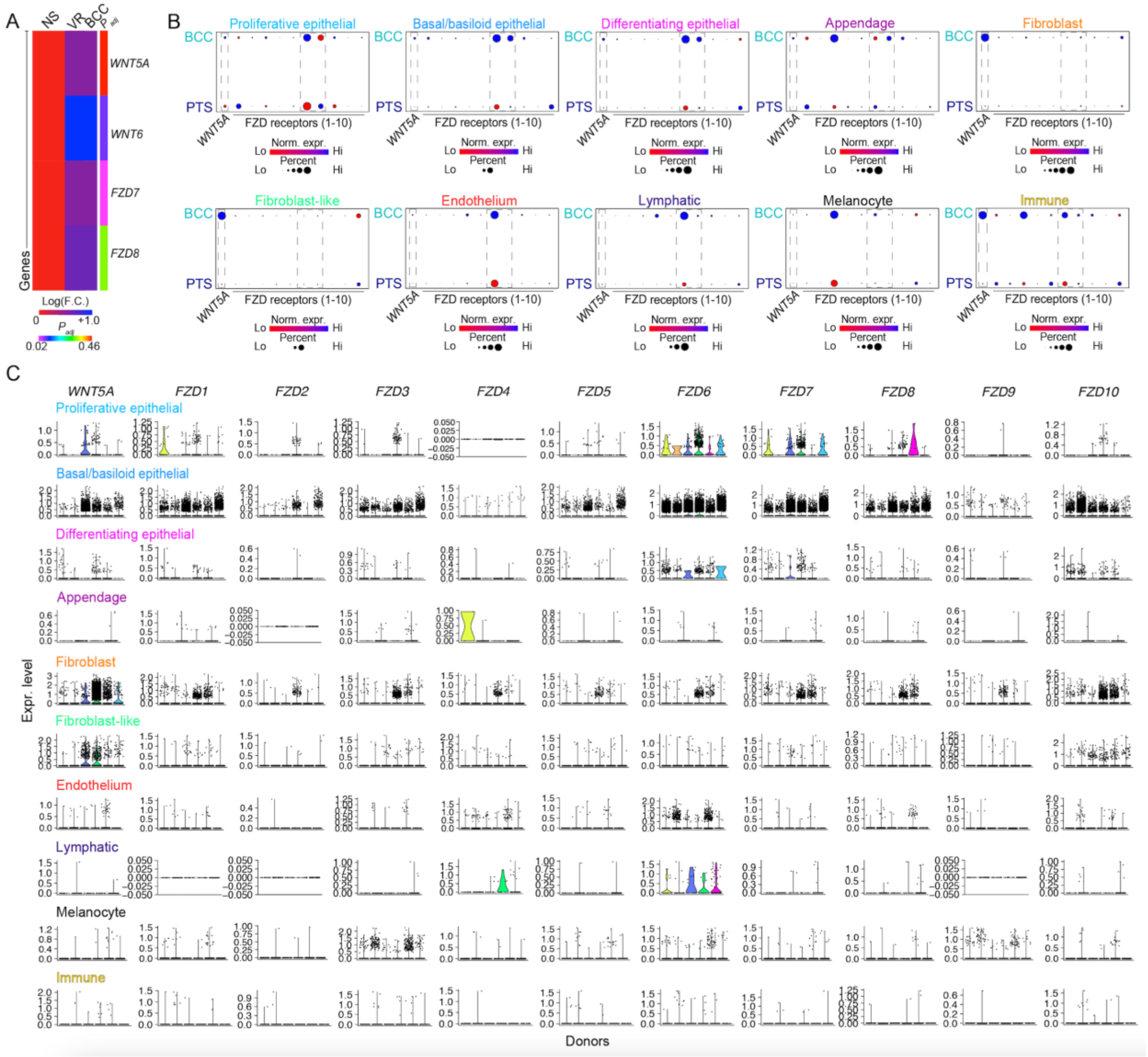

Supplementary Figure 9. Expression of ncWNT signaling pathway members. A. Heatmap of differentially expressed genes from non-canonical WNT signaling pathway in normal skin vs. vismodegib-resistant BCCs identified in Atwood *et al*. (*28*). Red – no change; blue – upregulation based on a Log fold change scale. Adjusted P-value scale is shown on the right based on a rainbow scale. Purple is most significant, and red is least significant. Genes of interest are on shown on the right. **B.** Dot plots demonstrating pseudo-bulk differential gene expression of genes in select cell types split by tissue type (peri-tumor skin vs. basal cell carcinoma). Blue – low expression of ncWNT ligand and receptors; red – high expression of non-canonical WNT ligand and receptors. Size of circle represents the percentage of cells expressing non-canonical WNT

ligand and receptors. **C.** Violin plots demonstrating expression level of non-canonical WNT ligand and receptors in select cell types split by tissue type (peri-tumor skin vs. basal cell carcinoma).

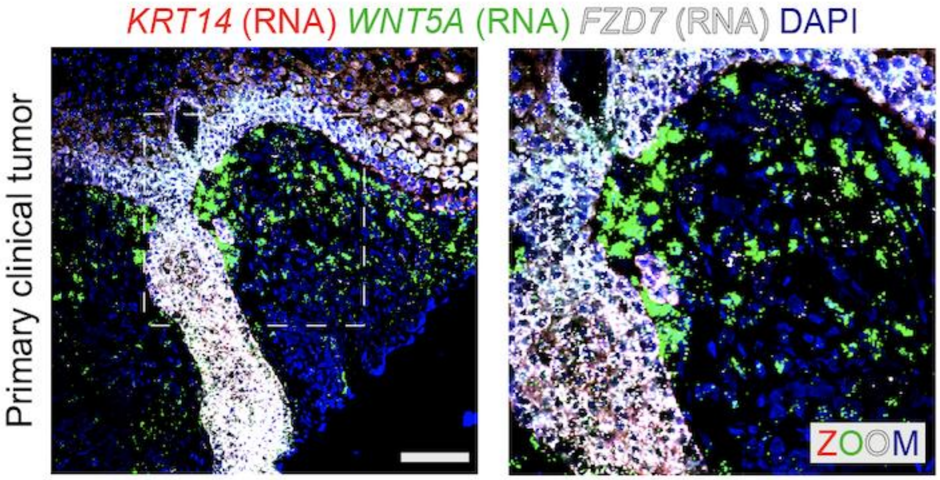

Supplementary Figure 10. Expression of non-canonical WNT ligand and receptor in basal cell carcinoma. RNA *in situ* hybridization staining of *WNT5A* (green) and *FZD7* (gray) show close spatial localization of *WNT5A*$^{+}$ FIBs and *FZD7*$^{+}$ epithelial cells in human primary clinical tumors. Inset shows magnified area in BCC nests. Tissues were counterstained with *KRT14* (red) and DAPI. Size bars: 100 µm.

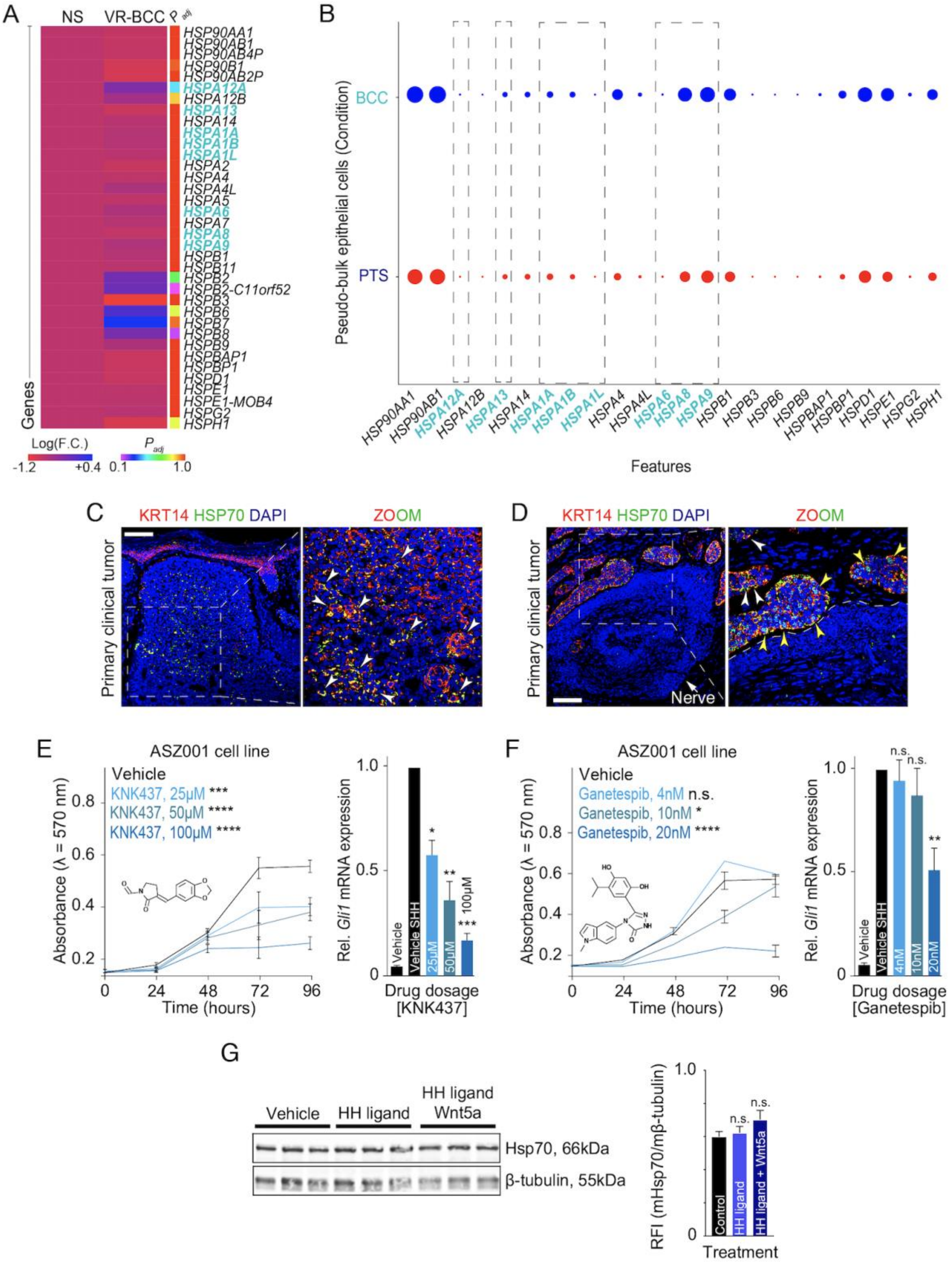
A
NS
VR-BCC
Genes
HSP90AA1
HSP90AB1
HSP90AB4P
HSP90B1
HSP90AB2P
HSPA12A
HSPA12B
HSPA13
HSPA14
HSPA1A
HSPA1B
HSPA1L
HSPA2
HSPA4
HSPA4L
HSPA5
HSPA6
HSPA7
HSPA8
HSPA9
HSPB1
HSPB11
HSPB2
HSPB2-C11orf52
HSPB3
HSPB6
HSPB7
HSPB8
HSPB9
HSPBAP1
HSPBP1
HSPD1
HSPE1
HSPE1-MOB4
HSPG2
HSPH1
Log(F.C.)
-1.2
+0.4
P_{adj}
0.1
1.0
B
Pseudo-bulk epithelial cells (Condition)
BCC
PTS
Features
Percent
Avg. expr.
Hi
Lo
C
KRT14 HSP70 DAPI
ZOOM
Primary clinical tumor
D
KRT14 HSP70 DAPI
ZOOM
Primary clinical tumor
Nerve
E
ASZ001 cell line
Vehicle
KNK437, 25μM ***
KNK437, 50μM ****
KNK437, 100μM ****
Absorbance (λ = 570 nm)
Time (hours)
Rel. Gli1 mRNA expression
Drug dosage [KNK437]
Vehicle
Vehicle SHH
25μM
50μM
100μM
F
ASZ001 cell line
Vehicle
Ganetespib, 4nM n.s.
Ganetespib, 10nM *
Ganetespib, 20nM ****
Absorbance (λ = 570 nm)
Time (hours)
Rel. Gli1 mRNA expression
Drug dosage [Ganetespib]
4nM
10nM
20nM
G
Vehicle
HH ligand
HH ligand Wnt5a
Hsp70, 66kDa
β-tubulin, 55kDa
RFI (mHsp70/mβ-tubulin)
Treatment
Control
HH ligand
HH ligand + Wnt5a
n.s.

Supplementary Figure 11. Heat shock protein-coding genes are upregulated in basal cell carcinoma. A. Heatmap of differentially expressed genes from ncWNT signaling pathway in normal skin vs. vismodegib-resistant BCCs identified in Atwood *et al*. (*28*). Red – gene downregulation; blue – gene upregulation. Adjusted P-value scale is based on a rainbow scale. Purple – most significant; red – least significant. HSP-coding genes are on shown on the right. **B.** Pseudo-bulk analysis of HSP-coding genes shows HSP-coding genes upregulated in human BCC epithelial cells. Gray – low average gene expression; blue – high average gene expression. HSP-coding genes belonging to the HSP70 family are demarcated and bolded in **(A)** and **(B)**. **C-D.** *In situ* expression of HSP70 protein shows distinct spatial localization in human primary BCC tumor. Inset shows magnified area in BCC nest. White arrows point at $HSP70^+$ epithelial cells in tumor nests. Size bars: 100 µm. **E-F.** HSP inhibitor KNK437 and ganetespib negatively impacts cell growth of ASZ001 murine cells (**E-F, right panel**) (Two-way ANOVA test; $^*P < 0.05$, $^{***}P < 0.001$, $^{****}P < 0.0001$, n.s. – not significant) and downregulate *Gli1* mRNA (**E-F, left panel**) *in vitro* in a concentration-dependent manner (Unpaired Student's two-tailed t test with Welch's correction; $^*P < 0.05$, $^{**} P < 0.005$, $^{***}P < 0.001$, n.s. – not significant). Experiments were repeated at least three times and data is represented as the mean of triplicates ± standard error of the mean (SEM). **G.** Western blot and quantification of RFI against Hsp70 in ASZ001 murine cells treated with vehicle, HH ligand, or HH ligand with recombinant Wnt5a protein. β-tubulin served as loading control. Mann Whitney test (n.s. – not significant).

Supplementary Table S1: Cell number quantification per individual before and after non-viable cell pruning and quality control (QC) filtering.

Patient ID	Sample type	Total cells before QC	Scrublet predicted doublets	Total cells after Scrublet and QC filtering
PTS-I	Peri-tumor skin	7,164	170	6,754
PTS-II	Peri-tumor skin	10,563	191	9,777
BCC-I	Primary basal cell carcinoma	10,058	671	9,387
BCC-II	Primary basal cell carcinoma	12,511	787	11,724
BCC-III	Primary basal cell carcinoma	7,094	145	6,712
BCC-IV	Primary basal cell carcinoma	8,829	168	8,569

Supplementary Table S2: 3'-droplet-enabled single-cell RNA-sequencing metrics – mean reads per cell.

Patient ID	Sample type	Mean reads per cell
PTS-I	Peri-tumor skin	36,983
PTS-II	Peri-tumor skin	30,012
BCC-I	Primary basal cell carcinoma	48,713
BCC-II	Primary basal cell carcinoma	33,074
BCC-III	Primary basal cell carcinoma	47,337
BCC-IV	Primary basal cell carcinoma	31,405

Supplementary Table S3: 3'-droplet-enabled single-cell RNA-sequencing metrics – median genes per cell.

Patient ID	Sample type	Median genes per cell
PTS-I	Peri-tumor skin	2,382
PTS-II	Peri-tumor skin	1,902
BCC-I	Primary basal cell carcinoma	3,515
BCC-II	Primary basal cell carcinoma	2,885
BCC-III	Primary basal cell carcinoma	2,315
BCC-IV	Primary basal cell carcinoma	1,983

REFERENCES AND NOTES

1. M. C. Cameron, E. Lee, B. P. Hibler, C. A. Barker, S. Mori, M. Cordova, K. S. Nehal, A. M. Rossi, Basal cell carcinoma: Epidemiology; pathophysiology; clinical and histological subtypes; and disease associations. *J. Am. Acad. Dermatol.* **80**, 303–317 (2019).

2. T. T. L. Nguyen, E. Tarapore, S. X. Atwood, in *Imaging Technologies and Transdermal Delivery in Skin Disorders* (John Wiley & Sons, 2019), pp. 83–103.

3. A. L. Chang, A. E. Oro, Initial assessment of tumor regrowth after vismodegib in advanced basal cell carcinoma. *Arch. Dermatol.* **148**, 1324–1325 (2012).

4. R. Y. Chow, T. M. Levee, G. Kaur, D. P. Cedeno, L. T. Doan, S. X. Atwood, MTOR promotes basal cell carcinoma growth through atypical PKC. *Exp. Dermatol.* **30**, 358–366 (2021).

5. R. Y. Chow, U. S. Jeon, T. M. Levee, G. Kaur, D. P. Cedeno, L. T. Doan, S. X. Atwood, PI3K promotes basal cell carcinoma growth through kinase-induced p21 degradation. *Front. Oncol.* **11**, 668247 (2021).

6. A. Sánchez-Danés, J.-C. Larsimont, M. Liagre, E. Muñoz-Couselo, G. Lapouge, A. Brisebarre, C. Dubois, M. Suppa, V. Sukumaran, V. D. Marmol, J. Tabernero, C. Blanpain, A slow-cycling LGR5 tumour population mediates basal cell carcinoma relapse after therapy. *Nature* **562**, 434–438 (2018).

7. S. X. Atwood, M. Li, A. Lee, J. Y. Tang, A. E. Oro, GLI activation by atypical protein kinase C ι/λ regulates the growth of basal cell carcinomas. *Nature* **494**, 484–488 (2013).

8. M. Eberl, D. Mangelberger, J. B. Swanson, M. E. Verhaegen, P. W. Harms, M. L. Frohm, A. A. Dlugosz, S. Y. Wong, Tumor architecture and notch signaling modulate drug response in basal cell carcinoma. *Cancer Cell* **33**, 229–243.e4 (2018).

9. F. Kuonen, N. E. Huskey, G. Shankar, P. Jaju, R. J. Whitson, K. E. Rieger, S. X. Atwood, K. Y. Sarin, A. E. Oro, Loss of primary cilia drives switching from hedgehog to Ras/MAPK pathway in resistant basal cell carcinoma. *J. Invest. Dermatol.* **139**, 1439–1448 (2019).

10. X. Zhao, T. Ponomaryov, K. J. Ornell, P. Zhou, S. K. Dabral, E. Pak, W. Li, S. X. Atwood, R. J. Whitson, A. L. S. Chang, J. Li, A. E. Oro, J. A. Chan, J. F. Kelleher, R. A. Segal, RAS/MAPK activation drives resistance to smo inhibition, metastasis, and tumor evolution in shh pathway-dependent tumors. *Cancer Res.* **75**, 3623–3635 (2015).

11. C. D. Yao, D. Haensel, S. Gaddam, T. Patel, S. X. Atwood, K. Y. Sarin, R. J. Whitson, S. McKellar, G. Shankar, S. Aasi, K. Rieger, A. E. Oro, AP-1 and TGFss cooperativity drives non-canonical Hedgehog signaling in resistant basal cell carcinoma. *Nat. Commun.* **11**, 5079 (2020).

12. R. J. Whitson, A. Lee, N. M. Urman, A. Mirza, C. Y. Yao, A. S. Brown, J. R. Li, G. Shankar, M. A. Fry, S. X. Atwood, E. Y. Lee, S. T. Hollmig, S. Z. Aasi, K. Y. Sarin, M. P. Scott, E. H. Epstein Jr, J. Y. Tang, A. E. Oro, Noncanonical hedgehog pathway activation through SRF-MKL1 promotes drug resistance in basal cell carcinomas. *Nat. Med.* **24**, 271–281 (2018).

13. K. Sasaki, T. Sugai, K. Ishida, M. Osakabe, H. Amano, H. Kimura, M. Sakuraba, K. Kashiwa, S. Kobayashi, Analysis of cancer-associated fibroblasts and the epithelial-mesenchymal transition in cutaneous basal cell carcinoma, squamous cell carcinoma, and malignant melanoma. *Hum. Pathol.* **79**, 1–8 (2018).

14. K. Lesack, C. Naugler, Morphometric characteristics of basal cell carcinoma peritumoral stroma varies among basal cell carcinoma subtypes. *BMC Dermatol.* **12**, 1 (2012).

15. S. H. Omland, E. E. Wettergren, S. Mollerup, M. Asplund, T. Mourier, A. J. Hansen, R. Gniadecki, Cancer associated fibroblasts (CAFs) are activated in cutaneous basal cell carcinoma and in the peritumoural skin. *BMC Cancer* **17**, 675 (2017).

16. P. A. Adegboyega, S. Rodriguez, J. McLarty, Stromal expression of actin is a marker of aggressiveness in basal cell carcinoma. *Hum. Pathol.* **41**, 1128–1137 (2010).

17. O. Abbas, J. E. Richards, M. Mahalingam, Fibroblast-activation protein: A single marker that confidently differentiates morpheaform/infiltrative basal cell carcinoma from desmoplastic trichoepithelioma. *Mod. Pathol.* **23**, 1535–1543 (2010).

18. L. Gonzalez-Silva, L. Quevedo, I. Varela, Tumor functional heterogeneity unraveled by scRNA-seq technologies. *Trends Cancer* **6**, 13–19 (2020).

19. E. Z. Macosko, A. Basu, R. Satija, J. Nemesh, K. Shekhar, M. Goldman, I. Tirosh, A. R. Bialas, N. Kamitaki, E. M. Martersteck, J. J. Trombetta, D. A. Weitz, J. R. Sanes, A. K. Shalek, A. Regev, S. A. McCarroll, Highly parallel genome-wide expression profiling of individual cells using nanoliter droplets. *Cell* **161**, 1202–1214 (2015).

20. T. Stuart, A. Butler, P. Hoffman, C. Hafemeister, E. Papalexi, W. M. Mauck III, Y. Hao, M. Stoeckius, P. Smibert, R. Satija, Comprehensive integration of single-cell data. *Cell* **177**, 1888–1902.e21 (2019).

21. I. Tirosh, B. Izar, S. M. Prakadan, M. H. Wadsworth II, D. Treacy, J. J. Trombetta, A. Rotem, C. Rodman, C. Lian, G. Murphy, M. Fallahi-Sichani, K. Dutton-Regester, J. R. Lin, O. Cohen, P. Shah, D. Lu, A. S. Genshaft, T. K. Hughes, C. G. K. Ziegler, S. W. Kazer, A. Gaillard, K. E. Kolb, A. C. Villani, C. M. Johannessen, A. Y. Andreev, E. M. van Allen, M. Bertagnolli, P. K. Sorger, R. J. Sullivan, K. T. Flaherty, D. T. Frederick, J. Jané-Valbuena, C. H. Yoon, O. Rozenblatt-Rosen, A. K. Shalek, A. Regev, L. A. Garraway, Dissecting the multicellular ecosystem of metastatic melanoma by single-cell RNA-seq. *Science* **352**, 189–196 (2016).

22. A. L. Ji, A. J. Rubin, K. Thrane, S. Jiang, D. L. Reynolds, R. M. Meyers, M. G. Guo, B. M. George, A. Mollbrink, J. Bergenstråhle, L. Larsson, Y. Bai, B. Zhu, A. Bhaduri, J. M. Meyers, X. Rovira-Clavé, S. T. Hollmig, S. Z. Aasi, G. P. Nolan, J. Lundeberg, P. A. Khavari, Multimodal analysis of composition and spatial architecture in human squamous cell carcinoma. *Cell* **182**, 497–514.e22 (2020).

23. C. Hafemeister, R. Satija, Normalization and variance stabilization of single-cell RNA-seq data using regularized negative binomial regression. *Genome Biol.* **20**, 296 (2019).

24. J. Liu, C. Gao, J. Sodicoff, V. Kozareva, E. Z. Macosko, J. D. Welch, Jointly defining cell types from multiple single-cell datasets using LIGER. *Nat. Protoc.* **15**, 3632–3662 (2020).

25. I. Korsunsky, N. Millard, J. Fan, K. Slowikowski, F. Zhang, K. Wei, Y. Baglaenko, M. Brenner, P. R. Loh, S. Raychaudhuri, Fast, sensitive and accurate integration of single-cell data with Harmony. *Nat. Methods* **16**, 1289–1296 (2019).

26. L. Zhang, Q. Nie, scMC learns biological variation through the alignment of multiple single-cell genomics datasets. *Genome Biol.* **22**, 10 (2021).

27. K. E. Yost, A. T. Satpathy, D. K. Wells, Y. Qi, C. Wang, R. Kageyama, K. L. McNamara, J. M. Granja, K. Y. Sarin, R. A. Brown, R. K. Gupta, C. Curtis, S. L. Bucktrout, M. M. Davis, A. L. S. Chang, H. Y. Chang, Clonal replacement of tumor-specific T cells following PD-1 blockade. *Nat. Med.* **25**, 1251–1259 (2019).

28. S. X. Atwood, K. Y. Sarin, R. J. Whitson, J. R. Li, G. Kim, M. Rezaee, M. S. Ally, J. Kim, C. Yao, A. L. S. Chang, A. E. Oro, J. Y. Tang, Smoothened variants explain the majority of drug resistance in basal cell carcinoma. *Cancer Cell* **27**, 342–353 (2015).

29. S. Bircan, O. Candir, N. Kapucoglu, S. Baspinar, The expression of p63 in basal cell carcinomas and association with histological differentiation. *J. Cutan. Pathol.* **33**, 293–298 (2006).

30. C. A. Orozco, N. Martinez-Bosch, P. E. Guerrero, J. Vinaixa, T. Dalotto-Moreno, M. Iglesias, M. Moreno, M. Djurec, F. Poirier, H. J. Gabius, M. E. Fernandez-Zapico, R. F. Hwang, C. Guerra, G. A. Rabinovich, P. Navarro, Targeting galectin-1 inhibits pancreatic cancer progression by modulating tumor-stroma crosstalk. *Proc. Natl. Acad. Sci. U.S.A.* **115**, E3769–E3778 (2018).

31. N. M. A. White, O. Masui, D. Newsted, A. Scorilas, A. D. Romaschin, G A Bjarnason, K. W. M. Siu, G. M. Yousef, Galectin-1 has potential prognostic significance and is implicated in clear cell renal cell carcinoma progression through the HIF/mTOR signaling axis. *Br. J. Cancer* **110**, 1250–1259 (2014).

32. M. Chetry, Y. Song, C. Pan, R. Li, J. Zhang, X. Zhu, Effects of galectin-1 on biological behavior in cervical cancer. *J. Cancer* **11**, 1584–1595 (2020).

33. A. Bolander, M. Agnarsdóttir, S. Strömberg, F. Ponten, P. Hesselius, M. Uhlen, M. Bergqvist, The protein expression of TRP-1 and galectin-1 in cutaneous malignant melanomas. *Cancer Genomics Proteomics* **5**, 293–300 (2008).

34. B. Van de Sande, C. Flerin, K. Davie, M. De Waegeneer, G. Hulselmans, S. Aibar, R. Seurinck, W. Saelens, R. Cannoodt, Q. Rouchon, T. Verbeiren, D. De Maeyer, J. Reumers, Y. Saeys, S. Aerts, A scalable SCENIC workflow for single-cell gene regulatory network analysis. *Nat. Protoc.* **15**, 2247–2276 (2020).

35. B. Han, Y. Qu, Y. Jin, Y. Yu, N. Deng, K. Wawrowsky, X. Zhang, N. Li, S. Bose, Q. Wang, S. Sakkiah, R. Abrol, T. W. Jensen, B. P. Berman, H. Tanaka, J. Johnson, B. Gao, J. Hao, Z. Liu, R. Buttyan, P. S. Ray, M.C. Hung, A. E. Giuliano, X. Cui, FOXC1 activates smoothened-independent hedgehog signaling in basal-like breast cancer. *Cell Rep.* **13**, 1046–1058 (2015).

36. R. Perez-Gomez, E. Haro, M. Fernandez-Guerrero, M. F. Bastida, M. A. Ros, Role of Hox genes in regulating digit patterning. *Int. J. Dev. Biol.* **62**, 797–805 (2018).

37. M. Reina-Campos, M. T. Diaz-Meco, J. Moscat, The dual roles of the atypical protein kinase Cs in cancer. *Cancer Cell* **36**, 218–235 (2019).

38. A. L. Kim, J. H. Back, S. C. Chaudhary, Y. Zhu, M. Athar, D. R. Bickers, SOX9 transcriptionally regulates mTOR-induced proliferation of basal cell carcinomas. *J. Invest. Dermatol.* **138**, 1716–1725 (2018).

39. H. S. Chahal, W. Wu, K. J. Ransohoff, L. Yang, H. Hedlin, M. Desai, Y. Lin, H.J. Dai, A. A. Qureshi, W.Q. Li, P. Kraft, D. A. Hinds, J. Y. Tang, J. Han, K. Y. Sarin, Genome-wide association study identifies 14 novel risk alleles associated with basal cell carcinoma. *Nat. Commun.* **7**, 12510 (2016).

40. V. Bergen, M. Lange, S. Peidli, F. A. Wolf, F. J. Theis, Generalizing RNA velocity to transient cell states through dynamical modeling. *Nat. Biotechnol.* **38**, 1408–1414 (2020).

41. V. Lopez-Pajares, K. Qu, J. Zhang, D. E. Webster, B. C. Barajas, Z. Siprashvili, B. J. Zarnegar, L. D. Boxer, E. J. Rios, S. Tao, M. Kretz, P. A. Khavari, A LncRNA-MAF:MAFB transcription factor network regulates epidermal differentiation. *Dev. Cell* **32**, 693–706 (2015).

42. B. Biehs, G. J. P. Dijkgraaf, R. Piskol, B. Alicke, S. Boumahdi, F. Peale, S. E. Gould, F. J. de Sauvage, A cell identity switch allows residual BCC to survive Hedgehog pathway inhibition. *Nature* **562**, 429–433 (2018).

43. C. Philippeos, S.B. Telerman, B. Oulès, A.O. Pisco, T.J. Shaw, R. Elgueta, G. Lombardi, R.R. Driskell, M. Soldin, M.D. Lynch, F.M. Watt, Spatial and single-cell transcriptional profiling identifies functionally distinct human dermal fibroblast subpopulations. *J. Invest. Dermatol.* **138**, 811–825 (2018).

44. C. F. Guerrero-Juarez, P. H. Dedhia, S. Jin, R. Ruiz-Vega, D. Ma, Y. Liu, K. Yamaga, O. Shestova, D. L. Gay, Z. Yang, K. Kessenbrock, Q. Nie, W. S. Pear, G. Cotsarelis, M. V. Plikus, Single-cell analysis reveals fibroblast heterogeneity and myeloid-derived adipocyte progenitors in murine skin wounds. *Nat. Commun.* **10**, 650 (2019).

45. R. R. Driskell, B. M. Lichtenberger, E. Hoste, K. Kretzschmar, B. D. Simons, M. Charalambous, S. R. Ferron, Y. Herault, G. Pavlovic, A. C. Ferguson-Smith, F. M. Watt, Distinct fibroblast lineages determine dermal architecture in skin development and repair. *Nature* **504**, 277–281 (2013).

46. R. M. Hughes, B. W. Simons, H. Khan, R. Miller, V. Kugler, S. Torquato, D. Theodros, M. C. Haffner, T. Lotan, J. Huang, E. Davicioni, S. S. An, R. C. Riddle, D. L.J. Thorek, I. P. Garraway, E. J. Fertig, J. T. Isaacs, W. N. Brennen, B. H. Park, P. J. Hurley, Asporin restricts mesenchymal stromal cell differentiation, alters the tumor microenvironment, and drives metastatic progression. *Cancer Res.* **79**, 3636–3650 (2019).

47. N. A. Evensen, Y. Li, C. Kuscu, J. Liu, J. Cathcart, A. Banach, Q. Zhang, E. Li, S. Joshi, J. Yang, P. I. Denoya, S. Pastorekova, S. Zucker, K. R. Shroyer, J. Cao, Hypoxia promotes colon cancer dissemination through up-regulation of cell migration-inducing protein (CEMIP). *Oncotarget* **6**, 20723–20739 (2015).

48. Z. H. Jiang, J. Peng, H.L. Yang, X.L. Fu, J.Z. Wang, L. Liu, J.N. Jiang, Y.F. Tan, Z.J. Ge, Upregulation and biological function of transmembrane protein 119 in osteosarcoma. *Exp. Mol. Med.* **49**, e329 (2017).

49. M. I. Love, W. Huber, S. Anders, Moderated estimation of fold change and dispersion for RNA-seq data with DESeq2. *Genome Biol.* **15**, 550 (2014).

50. F. de Sousa e Melo, L. Vermeulen, Wnt signaling in cancer stem cell biology. *Cancers (Basel)* **8**, 60 (2016).

51. S. Wang, M. L. Drummond, C. F. Guerrero-Juarez, E. Tarapore, A. L. MacLean, A. R. Stabell, S. C. Wu, G. Gutierrez, B. T. That, C. A. Benavente, Q. Nie, S. X. Atwood, Single cell transcriptomics of human epidermis identifies basal stem cell transition states. *Nat. Commun.* **11**, 4239 (2020).

52. M. Lange, V. Bergen, M. Klein, M. Setty, B. Reuter, M. Bakhti, H. Lickert, M. Ansari, J. Schniering, H. B. Schiller, D. Pe'er, F. J. Theis, CellRank for directed single-cell fate mapping. *Nat. Methods* **19**, 159–170 (2022).

53. X. Qiu, Q. Mao, Y. Tang, L. Wang, R. Chawla, H. A. Pliner, C. Trapnell, Reversed graph embedding resolves complex single-cell trajectories. *Nat. Methods* **14**, 979–982 (2017).

54. S. Jin, A. L. MacLean, T. Peng, Q. Nie, scEpath: Energy landscape-based inference of transition probabilities and cellular trajectories from single-cell transcriptomic data. *Bioinformatics* **34**, 2077–2086 (2018).

55. S. Jin, C. F. Guerrero-Juarez, L. Zhang, I. Chang, P. Myung, M. V. Plikus, Q. Nie, Inference and analysis of cell-cell communication using CellChat. *Nat. Commun.* **12**, 1088 (2021).

56. M. Pashirzad, M. Shafiee, F. Rahmani, R. B.-Rassouli, F. Hoseinkhani, M. Ryzhikov, M. M. Binabaj, M. R. Parizadeh, A. Avan, S. M. Hassanian, Role of Wnt5a in the pathogenesis of inflammatory diseases. *J. Cell. Physiol.* **232**, 1611–1616 (2017).

57. Y. S. Jung, H. Y. Lee, S. D. Kim, J. S. Park, J. K. Kim, P.-G. Suh, Y.-S. Bae, Wnt5a stimulates chemotactic migration and chemokine production in human neutrophils. *Exp. Mol. Med.* **45**, e27 (2013).

58. Y. Zhao, C. L. Wang, R. M. Li, T. Q. Hui, Y. Y. Su, Q. Yuan, X. D. Zhou, L. Ye, Wnt5a promotes inflammatory responses via nuclear factor κB (NF-κB) and mitogen-activated protein kinase (MAPK) pathways in human dental pulp cells. *J. Biol. Chem.* **292**, 4358 (2017).

59. P. C. Ikwegbue, P. Masamba, L. S. Mbatha, B. E. Oyinloye, A. P. Kappo, Interplay between heat shock proteins, inflammation and cancer: A potential cancer therapeutic target. *Am. J. Cancer Res.* **9**, 242–249 (2019).

60. A. N. Mirza, M. A. Fry, N. M. Urman, S. X. Atwood, J. Roffey, G. R. Ott, B. Chen, A. Lee, A. S. Brown, S. Z. Aasi, T. Hollmig, M. A. Ator, B. D. Dorsey, B. R. Ruggeri, C. A. Zificsak, M. Sirota, J. Y. Tang, A. Butte, E. Epstein, K. Y. Sarin, A. E. Oro, Combined inhibition of atypical PKC and histone deacetylase 1 is cooperative in basal cell carcinoma treatment. *JCI Insight* **2**, e97071 (2017).

61. S. C. Peterson, M. Eberl, A. N. Vagnozzi, A. Belkadi, N. A. Veniaminova, M. E. Verhaegen, C. K. Bichakjian, N. L. Ward, A. A. Dlugosz, S. Y. Wong, Basal cell carcinoma preferentially arises from stem cells within hair follicle and mechanosensory niches. *Cell Stem Cell* **16**, 400–412 (2015).

62. X. Bonilla, L. Parmentier, B. King, F. Bezrukov, G. Kaya, V. Zoete, V. B. Seplyarskiy, H. J. Sharpe, T. McKee, A. Letourneau, P. G. Ribaux, K. Popadin, N. Basset-Seguin, R. B. Chaabene, F. A. Santoni, M. A. Andrianova, M. Guipponi, M. Garieri, C. Verdan, K. Grosdemange, O. Sumara, M. Eilers, I. Aifantis, O. Michielin, F. J. de Sauvage, S. E. Antonarakis, S. I. Nikolaev, Genomic analysis identifies new drivers and progression pathways in skin basal cell carcinoma. *Nat. Genet.* **48**, 398–406 (2016).

63. H. J. Sharpe, G. Pau, G. J. Dijkgraaf, N. Basset-Seguin, Z. Modrusan, T. Januario, V. Tsui, A. B. Durham, A. A. Dlugosz, P. M. Haverty, R. Bourgon, J. Y. Tang, K. Y. Sarin, L. Dirix, D. C. Fisher, C. M. Rudin, H. Sofen, M. R. Migden, R. L. Yauch, F. J. de Sauvage, Genomic analysis of smoothened inhibitor resistance in basal cell carcinoma. *Cancer Cell* **27**, 327–341 (2015).

64. H. T. N. Tran, K. S. Ang, M. Chevrier, X. Zhang, N. Y. S. Lee, M. Goh, J. Chen, A benchmark of batch-effect correction methods for single-cell RNA sequencing data. *Genome Biol.* **21**, 12 (2020).

65. E. Sahai, I. Astsaturov, E. Cukierman, D. G. DeNardo, M. Egeblad, R. M. Evans, D. Fearon, F. R. Greten, S. R. Hingorani, T. Hunter, R. O. Hynes, R. K. Jain, T. Janowitz, C. Jorgensen, A. C. Kimmelman, M. G. Kolonin, R. G. Maki, R. S. Powers, E. Puré, D. C. Ramirez, R. Scherz-Shouval, M. H. Sherman, S. Stewart, T. D. Tlsty, D. A. Tuveson, F. M. Watt, V. Weaver, A. T. Weeraratna, Z. Werb, A framework for advancing our understanding of cancer-associated fibroblasts. *Nat. Rev. Cancer* **20**, 174–186 (2020).

66. K. L. Owen, N. K. Brockwell, B. S. Parker, JAK-STAT signaling: A double-edged sword of immune regulation and cancer progression. *Cancers (Basel)* **11**, 2002 (2019).

67. H. Techer, P. Pasero, The replication stress response on a narrow path between genomic instability and inflammation. *Front. Cell Dev. Biol.* **9**, 702584 (2021).

68. N. Andor, C. C. Maley, H. P. Ji, Genomic instability in cancer: Teetering on the limit of tolerance. *Cancer Res.* **77**, 2179–2185 (2017).

69. S. K. Calderwood, Heat shock proteins and cancer: Intracellular chaperones or extracellular signalling ligands? *Philos. Trans. R. Soc. Lond. B Biol. Sci.* **373**, 20160524 (2018).

70. L. Meng, C. Hunt, J. A. Yaglom, V. L. Gabai, M. Y. Sherman, Heat shock protein Hsp72 plays an essential role in Her2-induced mammary tumorigenesis. *Oncogene* **30**, 2836–2845 (2011).

71. C. R. Hunt, D. J. Dix, G. G. Sharma, R. K. Pandita, A. Gupta, M. Funk, T. K. Pandita, Genomic instability and enhanced radiosensitivity in Hsp70.1- and Hsp70.3-deficient mice. *Mol. Cell. Biol.* **24**, 899–911 (2004).

72. J. A. Yaglom, Y. Wang, A. Li, Z. Li, S. Monti, I. Alexandrov, X. Lu, M. Y. Sherman, Cancer cell responses to Hsp70 inhibitor JG-98: Comparison with Hsp90 inhibitors and finding synergistic drug combinations. *Sci. Rep.* **8**, 3010 (2018).

73. Y. L. Lai, H.H. Chang, M.J. Huang, K.H. Chang, W.H. Su, H.W. Chen, C.H. Chung, W.Y. Wang, L.H. Lin, Y.J. Chen, Combined effect of topical arsenic trioxide and radiation therapy on skin-infiltrating lesions of breast cancer—A pilot study. *Anticancer Drugs* **14**, 825–828 (2003).

74. A. Mielczarek-Lewandowska, M. L. Hartman, M. Czyz, Inhibitors of HSP90 in melanoma. *Apoptosis* **25**, 12–28 (2020).

75. J. Schindelin, I. Arganda-Carreras, E. Frise, V. Kaynig, M. Longair, T. Pietzsch, S. Preibisch, C. Rueden, S. Saalfeld, B. Schmid, J.Y. Tinevez, D. J. White, V. Hartenstein, K. Eliceiri, P. Tomancak, A. Cardona, Fiji: An open-source platform for biological-image analysis. *Nat. Methods* **9**, 676–682 (2012).

76. S. L. Wolock, R. Lopez, A. M. Klein, Scrublet: Computational identification of cell doublets in single-cell transcriptomic data. *Cell Syst.* **8**, 281–291.e9 (2019).

77. J. Fan, N. Salathia, R. Liu, G. E. Kaeser, Y. C. Yung, J. L. Herman, F. Kaper, J.B. Fan, K. Zhang, J. Chun, P. V. Kharchenko, Characterizing transcriptional heterogeneity through pathway and gene set overdispersion analysis. *Nat. Methods* **13**, 241–244 (2016).

78. J. D. Welch, V. Kozareva, A. Ferreira, C. Vanderburg, C. Martin, E. Z. Macosko, Single-cell multi-omic integration compares and contrasts features of brain cell identity. *Cell* **177**, 1873–1887.e17 (2019).

79. R. Takahashi, A. Grzenda, T. F. Allison, J. Rawnsley, S. J. Balin, S. Sabri, K. Plath, W. E. Lowry, Defining transcriptional signatures of human hair follicle cell states. *J. Invest. Dermatol.* **140**, 764–773.e4 (2020).

80. A. Subramanian, P. Tamayo, V. K. Mootha, S. Mukherjee, B. L. Ebert, M. A. Gillette, A. Paulovich, S. L. Pomeroy, T. R. Golub, E. S. Lander, J. P. Mesirov, Gene set enrichment analysis: A knowledge-based approach for interpreting genome-wide expression profiles. *Proc. Natl. Acad. Sci. U.S.A.* **102**, 15545–15550 (2005).

81. J. Alquicira-Hernandez, J. E. Powell, Nebulosa recovers single cell gene expression signals by kernel density estimation. *Bioinformatics*, **37**, 2485–2487 (2021).

82. P. D. Thomas, M. J. Campbell, A. Kejariwal, H. Mi, B. Karlak, R. Daverman, K. Diemer, A. Muruganujan, A. Narechania, PANTHER: A library of protein families and subfamilies indexed by function. *Genome Res.* **13**, 2129–2141 (2003).

83. D. W. Huang, B. T. Sherman, Q. Tan, J. Kir, D. Liu, D. Bryant, Y. Guo, R. Stephens, M. W. Baseler, H. C. Lane, R. A. Lempicki, DAVID bioinformatics resources: Expanded annotation database and novel algorithms to better extract biology from large gene lists. *Nucleic Acids Res.* **35**, W169–W175 (2007).

84. M. V. Kuleshov, M. R. Jones, A. D. Rouillard, N. F. Fernandez, Q. Duan, Z. Wang, S. Koplev, S. L. Jenkins, K. M. Jagodnik, A. Lachmann, M. G. McDermott, C. D. Monteiro, G. W. Gundersen, A.

Ma'ayan, Enrichr: A comprehensive gene set enrichment analysis web server 2016 update. *Nucleic Acids Res.* **44**, W90–W97 (2016).

85. E. Y. Chen, C. M. Tan, Y. Kou, Q. Duan, Z. Wang, G. V. Meirelles, N. R. Clark, A. Ma'ayan, Enrichr: Interactive and collaborative HTML5 gene list enrichment analysis tool. *BMC Bioinformatics* **14**, 128 (2013).

86. C. Trapnell, D. Cacchiarelli, J. Grimsby, P. Pokharel, S. Li, M. Morse, N. J. Lennon, K. J. Livak, T. S. Mikkelsen, J. L. Rinn, The dynamics and regulators of cell fate decisions are revealed by pseudotemporal ordering of single cells. *Nat. Biotechnol.* **32**, 381–386 (2014).

87. H. M. Zhang, T. Liu, C.J. Liu, S. Song, X. Zhang, W. Liu, H. Jia, Y. Xue, A. Y. Guo, AnimalTFDB 2.0: A resource for expression, prediction and functional study of animal transcription factors. *Nucleic Acids Res.* **43**, D76–D81 (2015).

88. S. Jin, C. F. Guerrero-Juarez, L. Zhang, I. Chang, R. Ramos, C.H. Kuan, P. Myung, M. V. Plikus, Q. Nie, Inference and analysis of cell-cell communication using CellChat. *Nat. Commun.* **12**, 1088 (2021).

89. S. Aibar, C. B. González-Blas, T. Moerman, V. A. Huynh-Thu, H. Imrichova, G. Hulselmans, F. Rambow, J.C. Marine, P. Geurts, J. Aerts, J. van den Oord, Z. K. Atak, J. Wouters, S. Aerts, SCENIC: Single-cell regulatory network inference and clustering. *Nat. Methods* **14**, 1083–1086 (2017).
